# Characterization of the dual regulation by a c-di-GMP riboswitch Bc1 with a long expression platform from *Bacillus thuringiensis*

**DOI:** 10.1128/spectrum.00450-24

**Published:** 2024-05-31

**Authors:** Lu Liu, Dehua Luo, Yongji Zhang, Dingqi Liu, Kang Yin, Qing Tang, Shan-Ho Chou, Jin He

**Affiliations:** 1 National Key Laboratory of Agricultural Microbiology & Hubei Hongshan Laboratory, College of Life Science and Technology, Huazhong Agricultural University, Wuhan, China; South China Sea Institute of Oceanology Chinese Academy of Sciences, Guangzhou, Guangdong, China

**Keywords:** *Bacillus cereus *group, cyclic di-GMP riboswitch, dual terminator, dual regulation, SHAPE-MaP, Zur (zinc uptake regulator)

## Abstract

A riboswitch generally regulates the expression of its downstream genes through conformational change in its expression platform (EP) upon ligand binding. The cyclic diguanosine monophosphate (c-di-GMP) class I riboswitch Bc1 is widespread and conserved among *Bacillus cereus* group species. In this study, we revealed that Bc1 has a long EP with two typical ρ-independent terminator sequences 28 bp apart. The upstream terminator T1 is dominant *in vitro*, while downstream terminator T2 is more efficient *in vivo*. Through mutation analysis, we elucidated that Bc1 exerts a rare and incoherent “transcription-translation” dual regulation with T2 playing a crucial role. However, we found that Bc1 did not respond to c-di-GMP under *in vitro* transcription conditions, and the expressions of downstream genes did not change with fluctuation in intracellular c-di-GMP concentration. To explore this puzzle, we conducted SHAPE-MaP and confirmed the interaction of Bc1 with c-di-GMP. This shows that as c-di-GMP concentration increases, T1 unfolds but T2 remains almost intact and functional. The presence of T2 masks the effect of T1 unwinding, resulting in no response of Bc1 to c-di-GMP. The high Shannon entropy values of EP region imply the potential alternative structures of Bc1. We also found that zinc uptake regulator can specifically bind to the dual terminator coding sequence and slightly trigger the response of Bc1 to c-di-GMP. This work will shed light on the dual-regulation riboswitch and enrich our understanding of the RNA world.

**IMPORTANCE:**

In nature, riboswitches are involved in a variety of metabolic regulation, most of which preferentially regulate transcription termination or translation initiation of downstream genes in specific ways. Alternatively, the same or different riboswitches can exist in tandem to enhance regulatory effects or respond to multiple ligands. However, many putative conserved riboswitches have not yet been experimentally validated. Here, we found that the c-di-GMP riboswitch Bc1 with a long EP could form a dual terminator and exhibit non-canonical and incoherent “transcription-translation” dual regulation. Besides, zinc uptake regulator specifically bound to the coding sequence of the Bc1 EP and slightly mediated the action of Bc1. The application of SHAPE-MaP to the dual regulation mechanism of Bc1 may establish the foundation for future studies of such complex untranslated regions in other bacterial genomes.

## INTRODUCTION

Cyclic diguanosine monophosphate (c-di-GMP) is a global nucleotide second messenger molecule in bacteria (1, 2). It has been extensively found to regulate bacterial motility (3–6), biofilm formation (7–9), virulence (10, 11), cell cycle and differentiation (12, 13), primary and secondary metabolism (14–17), and other biological processes. The versatility of c-di-GMP in different physiological activities is due to its ability to adopt multiple conformations (18) to interact with diverse downstream receptors (19, 20), which can be divided into protein (21–29) and RNA receptors (30–33).

RNA receptors are the so-called riboswitches, cis-regulatory elements located in the 5′ untranslated region (UTR) of bacterial transcripts (34, 35); when a specific ligand binds to its aptamer domain (AD), it induces conformational change in the expression platform (EP), thereby regulating the expression of downstream genes (20, 36). c-di-GMP riboswitches can be divided into class I (c-di-GMP-I) (33, 37, 38) and class II (c-di-GMP-II) based on structure, sequence conservation, and regulatory mode (39–43). The former generally controls the transcription termination or translation initiation of downstream genes by forming terminators/anti-terminators or sequesters/anti-sequesters (44–46), while the latter affects the RNA processing of downstream genes by controlling the self-splicing activity of ribozymes or the access of nuclease cleavage sites (32, 37, 47). The c-di-GMP-I riboswitch is further divided into type I and type II; c-di-GMP type I riboswitch, which can form a GNRA (G: guanine; N: adenine, guanine, cytosine, uracil; R: adenine, guanine; A: adenine) tetraloop and a corresponding tetraloop receptor, is usually located upstream of Genes related to Environment, Membrane and Motility; therefore, it is also called GEMM motif (33, 48, 49). The c-di-GMP type II riboswitch usually forms a GYRA (G: guanine; Y: cytosine, uracil; R: adenine, guanine; A: adenine) tetraloop, but it does not form a corresponding tetraloop receptor (33, 39).

There are numerous predicted c-di-GMP riboswitches (37, 38, 43, 50–53), but only a few have been experimentally verified (54–57). Research on c-di-GMP riboswitches has progressed slowly, primarily due to the variable structure and function of EP (58, 59).

Bc1 is a putative c-di-GMP-I riboswitch widely distributed in the genomes of bacteria of the *B. cereus* group (60). It is frequently found upstream of genes encoding methyl-accepting chemotaxis protein (MCP). Since its discovery in 2008, Bc1 has been shown to turn on the transcription of downstream reporter genes in *Bacillus subtilis* (33). However, little is known about its structures and regulatory mechanisms of its downstream genes in native genetic contexts. In recent years, selective 2′-hydroxyl acylation analyzed by primer extension and mutational profiling (SHAPE-MaP) has become a powerful method for characterizing regulatory RNA elements such as riboswitches, as it excels in accurate prediction and rapid identification of RNA structures, helping disclose their regulatory mechanisms (61–64). Here, through multiple *in vitro* and *in vivo* assays, combined with SHAPE-MaP, we confirmed that the c-di-GMP-I riboswitch Bc1 of *Bacillus thuringiensis* BMB171 strain (65) could regulate its target genes through a complex “transcription-translation” dual regulation mechanism. A zinc uptake regulator (Zur, BMB171_RS21350) was also found to bind directly to the Bc1 dual terminator coding sequence and exhibited a minor effect on Bc1’s response to c-di-GMP.

## RESULTS

### Bc1 was widely distributed and conserved among *Bacillus cereus* group

Our previous study found that 98.7% of 155 sequenced genomes from *B. cereus* group strains display Bc1-*mcp* gene architecture (Fig. S1) (60). According to the position of the *mcp* gene in the BMB171 genome and the nomenclature of *B. subtilis* MCP (66, 67), we named the *mcp* gene (*BMB171_RS02220*) downstream of Bc1 as *mcpE*.

**FIG 1:**
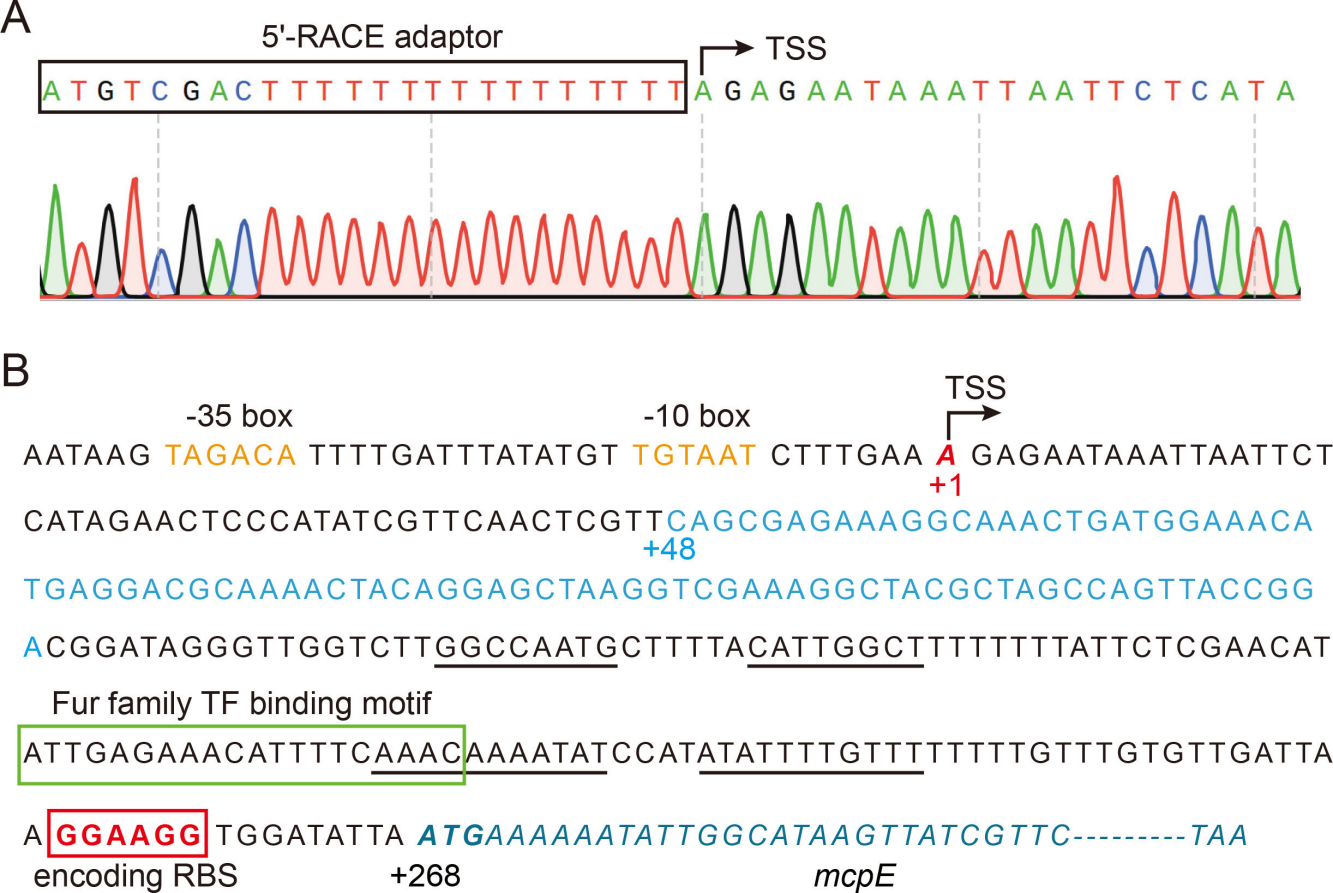
Analysis of the 5' UTR coding sequence of *mcpE*. (**A**) TSS mapping of *mcpE* through 5′-RACE assay. Black box indicates the 5′-RACE linker sequence, which contains poly(T)_16_, and black arrow refers to the +1 base A of the TSS. (**B**) Coding sequence of the 5′ UTR of the *mcpE* transcript. Red bold italic letter A indicates TSS, also highlighted with +1, TSS and black arrow; Orange indicates the −10 box and −35 box of the *mcpE* promoter; Light blue indicates the Bc1 AD coding sequence; Black underline represents the predicted hairpin sequence of both terminators; Green box represents the predicted Ferric uptake regulator (Fur) family TFs binding motif; Red box and red bold fonts emphasize the coding sequence of the RBS; and blue italics represent the *mcpE* gene sequence. See also Fig. S1 and S2.

To analyze the 5′ UTR of Bc1-*mcpE*, we conducted 5′ Rapid Amplification of cDNA Ends (5′-RACE) experiments to identify the transcription start site (TSS) of *mcpE*. Nucleotide A immediately following the 5′-RACE linker was considered a TSS (+1) and is located 267 bp upstream of Adenine-Uracil-Guanine (AUG), the translation start site of the *mcpE* transcript (Fig. 1A). We also predicted the −10 box (TAGACA) and the −35 box (TGTAAT) promoter elements upstream of the TSS (Fig. 1B). Therefore, the region from nucleotides + 1 to +267 was the 5′ UTR of the *mcpE* transcript, with nucleotides + 48 to +132 being the coding sequence for Bc1 AD (GEMM motif) (colored in light blue in Fig. 1B).

To further analyze the conservation of Bc1, we compared the sequence from the beginning of Bc1 AD to the start codon AUG (+48 to +267) of the *mcpE* transcript with the 152 bp Bc2 (44) from BMB171 strain, 200 bp Vc1 (68) and 224 bp Vc2 (69, 70) from *Vibrio cholerae*, which represent the three reported c-di-GMP-I riboswitches (Fig. S2A). Bc1 AD was found to be more conserved than EP, consistent with the characteristics of the riboswitches. The similarity between Bc1 and Vc2 was higher, followed by Vc1 and Bc2.

Taking into account the conservation of the sequence (Fig. S1) and the putative structure of the c-di-GMP-I riboswitch (33), we modeled the secondary structure of Bc1 AD, as shown in Fig. S2B, where Bc1 has a GAAA tetraloop and a tetraloop receptor, typical of c-di-GMP-I type I riboswitch. We predicted that nucleotides G11, A38, and C81 in Bc1, which correspond to nucleotides G20, A47, and C92 in Vc2, may play an important role in the interaction of Bc1 with c-di-GMP.

### Bc1 containing an intrinsic dual terminator inhibited downstream genes expressions

Unlike Bc2, Bc1 has a longer EP, up to 220 bp between AD to the downstream start codon AUG. As mentioned previously, the overall similarity between Bc1 and Bc2 was low (Fig. S2A), implying that the action mode of these two c-di-GMP-I riboswitches may differ significantly in the BMB171 strain. We wondered Bc1 may exhibit a more complex regulatory mechanism for its downstream genes.

**FIG 2:**
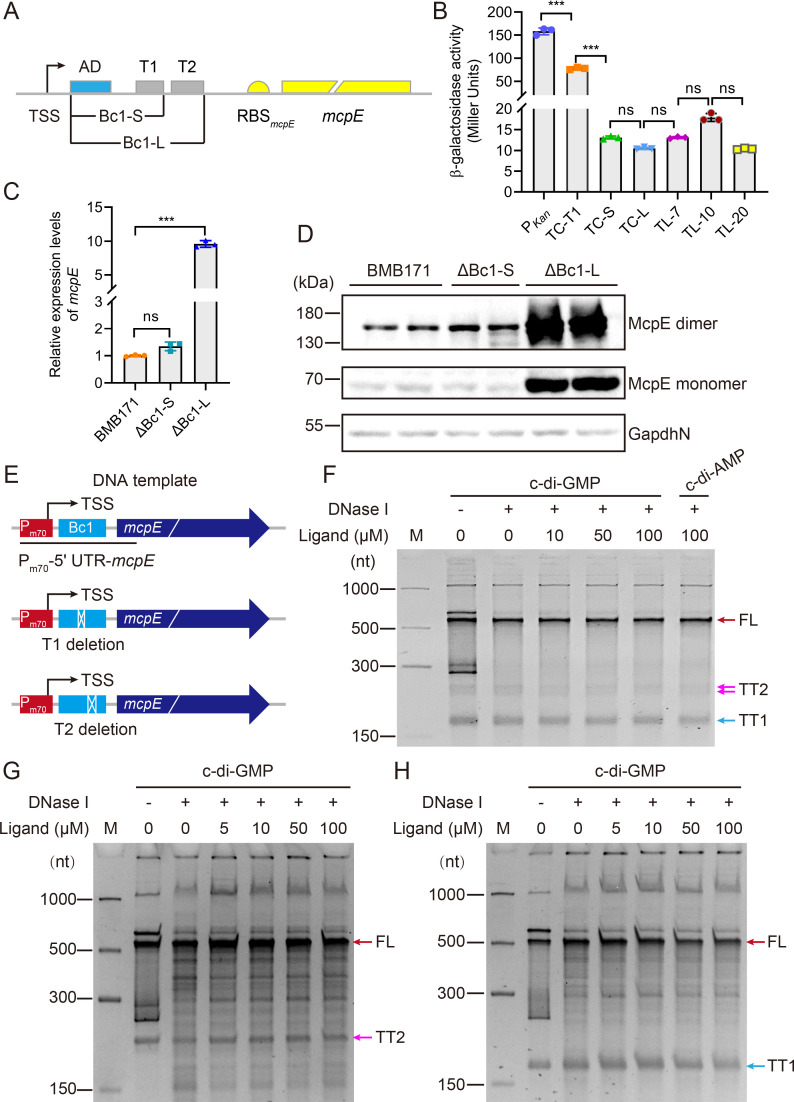
Bc1 forms an intrinsic dual terminator and inhibits downstream gene expressions. (A) Schematic diagram of the Bc1-*mcpE* gene structure. The coding sequence of Bc1 AD is shown in blue, and its dual terminator is shown in grey, with AD, T1 and T2 labeled next to it. The short Bc1-S (containing the coding sequence from Bc1 AD to T1) and long Bc1-L (containing the coding sequence from Bc1 AD to T2) regions are annotated separately. Sequences encoding the RBS and *mcpE* gene are shown in yellow. (B) β-Galactosidase activity assays of BMB171 strain containing different transcriptional and translational fusion reporter constructs. To assist the reader, we abbreviate transcriptional fusion as TC and translational fusion as TL. P*_Kan_* indicates a reporter construct, in which *lacZ* is transcriptionally fused to the promoter P*_Kan_*. TC-T1, TC-S, TC-L denote constructs, in which *lacZ* is transcriptionally fused to P*_Kan_* and varying lengths of 5′ UTR coding sequences up to terminator T1, two terminators, or the *mcpE* RBS, respectively. TL-7, TL-10 and TL-20 denote constructs in which *lacZ* is translationally fused to P*_Kan_*, the entire 5′ UTR coding sequence, and the first 7, 10 or 20 codons of the *mcpE* gene, respectively. (C) RT-qPCR assay of *mcpE* transcript levels after deleting the single terminator T1 or the Bc1 dual terminator. Values in *B* and *C* are mean ± standard deviation of three biological replicates, and the significance of the difference was analyzed by one-way ANOVA in GraphPad Prism 8.0.6 with Bonferroni correction, ns means no significance, *P* > 0.05; *P < 0.05; **P < 0.01; ***P < 0.001. (D) Western blot assay of McpE protein levels after deleting the single terminator T1 or the Bc1 dual terminator. The target 6× His-McpE-6× His monomer is approximately 68.6 kDa, and its dimer is approximately 137.2 kDa. GapdhN (BMB171_RS04385, NADP-dependent glyceraldehyde-3-phosphate dehydrogenase) with a C terminal 6× His tag was used as a control loading protein with a total size of nearly 53.1 kDa. (E) Schematic diagram of the DNA template used in *in vitro* transcription termination assays in (F–H). The wild-type template is shown at the top. The native promoter P_m70_ of *mcpE* is shown in red, the riboswitch Bc1 in light blue, and the *mcpE* gene in dark blue and italic. Deletion of terminator T1 (medium) or T2 (bottom) is indicated by white cross boxes. (F), (G) and (H) *In vitro* transcription termination assays using wild-type template, or mutant template with T1 or T2 deletion, respectively. M denotes the ssRNA marker, whose bands are indicated on the left as 1000, 500, 300, and 150 nt. FL transcripts are indicated by red arrows, and truncated transcripts terminated by T1 (TT1) or T2 (TT2) are indicated in blue or carmine arrows, respectively. Working concentrations of c-di-GMP are 0, 5, 10, 50 and 100 µM. 100 µM c-di-AMP represents the control group. See also Fig. S3.

We used CoFold database to analyze potential terminators or sequesters in the Bc1 EP. As expected, two sets of inverted palindromic sequences that are closely complementary to each other are predicted downstream of the Bc1 AD (Fig. 1B), possibly forming two hairpin structures. Further, the two hairpins are far away from the ribosome-binding site (RBS), and are both followed by a string of poly(U), in line with the characteristic of a ρ-independent transcription terminator. Therefore, we speculated that these two structures may serve as transcription terminators to regulate the read-through of downstream genes, and named them Terminator 1 (T1, +150 to +178) and Terminator 2 (T2, +207 to +237) (see Fig. 1B for the coding sequence and Fig. 2A for the gene structure). The distance between T1 and T2 is 28 bp.

To test whether T1 or T2 could serve as a transcription terminator, we performed *in vitro* transcription termination assays using the native promoter P_m70_ (−60 to +10) of Bc1-*mcpE* (Fig. 2E and F; Fig. S3D). If the RNA polymerase (RNAP) reads through the template or is depolymerized by T1 or T2, the resulting transcript of full-length (FL), transcript terminated at T1 (TT1), or transcript terminated at T2 (TT2) will be 496 nt, 178 nt, or 237 nt in size, respectively. From the results, we observed a distinct band around 500 nt and a band close to 150 nt (Fig. 2F), approaching the size of FL and TT1, respectively. We repeated the assays by changing native P_m70_ to the strong promoter J23119 and obtained similar results (Fig. S3E). Additionally, we noticed two narrow and weak bands, similar in size to TT2 (Fig. 2F; Fig. S3E). This implied that T1 was an efficient terminator under *in vitro* transcription conditions. After deleting T1 from the template (Fig. 2E and G), we observed a FL band of approximately 467 nt and a truncated band of approximately 208 nt consistent with the size of TT2. After deleting T2 alone from the template (Fig. 2E and H), we observed a FL band of about 465 nt, along with a truncated band of approximately 178 nt, which was similar in size to TT1. These results indicate that both T1 and T2 are effective terminators *in vitro*.

To investigate the roles of T1 and T2 in a natural genome context, we deleted the coding sequence from the Bc1 AD to the T1 poly(T) trail (named Bc1-S, +48 to +182) (Fig. 2A) in BMB171 genome, or the coding sequence from the Bc1 AD to the T2 poly(T) trail (named Bc1-L, +48 to +250) (Fig. 2A), to obtain the ΔBc1-S and ΔBc1-L mutants (Fig. 2C). After deletion of Bc1-S, the transcriptional level of *mcpE* increased only 1.2-fold, but after deletion of Bc1-L, it increased approximately 10-fold. We also examined the expression of McpE protein (Fig. 2D). The results indicate that after Bc1-S deletion, McpE protein level remained almost unchanged, whereas after Bc1-L deletion, McpE significantly accumulated and formed more McpE dimers (Fig. 2D). Taken together, in the genomic context, upstream T1 seemed to be bypassed, while the presence of downstream T2 dominated, strongly repressing the transcription of downstream genes.

To further confirm the regulatory role of T1 and T2 *in vivo*, we conducted the reporter assays utilizing the *lacZ* gene. We found that the transcriptional activity of the exogenous constitutive promoter P*_Kan_* from *B. subtilis* strain Bs168 did not change among the Δ*2dgc* mutant with low c-di-GMP concentration, the starting strain BMB171 with normal c-di-GMP concentration, and the Δ*3pde* mutant with high c-di-GMP concentration (Fig. S3B) (71). This indicates that P*_Kan_* does not respond to c-di-GMP. As the strains containing the reporter plasmids with *lacZ* fused to the *mcpE* 5′ UTR coding sequence did not show any β-galactosidase activity, suggesting that there is no alternative promoter in 5′ UTR. Therefore, the P*_Kan_* promoter and 5′ UTR are appropriate for combining to test the functions of T1 and T2. We then fused 5′ UTRs coding sequences of different lengths (including AD, the two terminators of Bc1, and the RBS of the *mcpE* transcript) and part of the *mcpE* coding sequence (CDS) with P*_Kan_* and *lacZ* to construct a series of different reporter constructs (Fig. S3A). To assist the reader, we abbreviated transcriptional fusion as TC and translational fusion as TL. The results show that β-galactosidase activity is reduced two-fold when the transcriptionally fused 5′ UTR contains only AD and T1 (TC-T1) (Fig. 2B). However, in the transcriptional fusion construct containing AD, T1 and T2 without *mcpE* RBS (TC-short, TC-S) (Fig. S3A), β-galactosidase activity was decreased by at least 10-fold (Fig. 2B; Fig. S3C), indicating that T1 and T2 together robustly repressed the transcription of *lacZ*, with T2 being the stronger terminator. To evaluate the effect of RBS presence on dual terminator-repressed transcription, we added a “TAA” stop codon after the coding sequence containing the entire 5′ UTR of AD, T1, T2, and RBS, and then fused it transcriptionally to *lacZ* (TC-long, TC-L) (Fig. S3A). The results demonstrates that the presence of RBS has little effect on the transcriptional repression mediated by the dual terminator (Fig. 2B; Fig. S3C).

**FIG 3:**
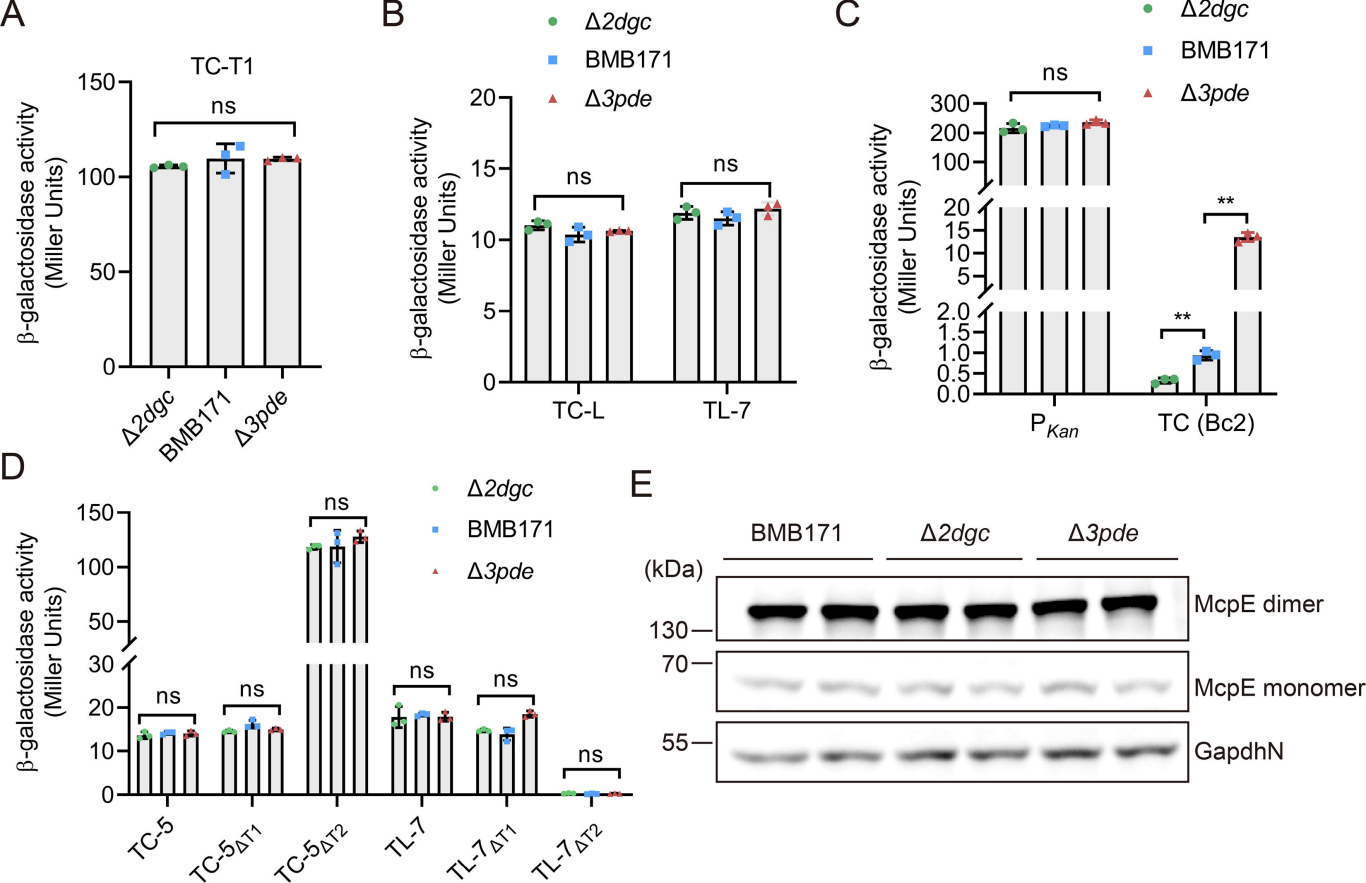
Bc1 has little effect on the expressions of downstream genes under different intracellular c-di-GMP concentrations. (**A**) β-Galactosidase activity assay of Δ*2dgc*, BMB171, and Δ*3pde* strains containing the transcriptional fusion construct TC-T1. (**B**) β-Galactosidase activity assay of Δ*2dgc*, BMB171, and Δ*3pde* strains containing transcriptional fusion TC-L and translational fusion TL-7 constructs. (**C**) β-Galactosidase activity assay of Δ*2dgc*, BMB171, and Δ*3pde* strains harboring transcriptional fusion constructs P*_Kan_* or TC (Bc2) (the coding sequence of 5′ UTR of the gene *cap* downstream of Bc2 is transcriptionally fused to P*_Kan_* and *lacZ*). (**D**) β-Galactosidase activity assays of Δ*2dgc*, BMB171, and Δ*3pde* strains harboring transcriptional fusion constructs TC-5, TC-5_ΔT1_, TC-5_ΔT2_, and the translational fusion constructs TL-7, TL-7_ΔT1_, TL-7_ΔT2_. ΔT1, ΔT2 indicate that the test sequence is fused with T1 or T2 deletion alone. (**E**) Western blot assay of McpE protein levels in Δ*2dgc*, BMB171, and Δ*3pde* strains. GapdhN was served as a control loading protein. Values in A, B, C and D are mean ± standard deviation of three biological replicates, and the significance of the difference was analyzed by two-way ANOVA in GraphPad Prism 8.0.6 with Bonferroni correction. ns means no significance, *P* > 0.05; *P < 0.05; **P < 0.01; ***P < 0.001.

Next, to examine whether Bc1 also functions at the translational level, we compared the β-galactosidase activity of constructs, in which *lacZ* were translationally fused to *mcpE* coding sequences of varying lengths. We found that the construct containing the first two codons of *mcpE* (TL-2) had almost no β-galactosidase activity (Fig. S3C), whereas the construct containing the first seven codons (TL-7) inhibited β-galactosidase activity by approximately 15-fold, which was not significantly different from transcriptional inhibition by the Bc1 dual-terminator (TC-L) (Fig. 2B). Further extension of codons to 10 (TL-10) or 20 (TL-20) did not significantly alter β-galactosidase activity (Fig. 2B), indicating that under this physiological condition, Bc1 mainly inhibits downstream genes at the transcriptional level. We further investigated the role of T1 and T2 in Bc1 regulation (Fig. 3D). It indicates that deletion of T1 had no significant effect on the transcriptional (TC-5_ΔT1_) or translational (TL-7_ΔT1_) activity of the reporter gene. However, after deleting T2, the transcriptional activity (TC-5_ΔT2_) of the reporter gene was significantly increased, while the translational activity (TL-7_ΔT2_) was completely abolished. These results suggest that Bc1 has a dual regulation of “transcription-translation” with opposite effects, and that T2 is responsible for this regulatory mechanism, while T1 has little effect.

According to the above experiments, we concluded that Bc1 could form a tandem intrinsic terminator, robustly repressing the expression of downstream genes. *In vitro*, T1 seems to be more effective in transcription termination. However, under physiological conditions, the upstream terminator T1 was weak, while the downstream terminator T2 played a major role in the incoherent “transcription-translation” dual regulation of Bc1.

### Bc1 hardly affected the expression of downstream genes

To evaluate whether Bc1 can respond to c-di-GMP or regulate its downstream genes under physiological conditions, we examined the expression of McpE in Δ*2dgc*, BMB171 and Δ*3pde* strains. Unexpectedly, we did not see any difference in McpE levels (Fig. 3E). We then introduced various transcriptional and translational fusion reporter constructs (Fig. S3A) into the Δ*2dgc*, BMB171 and Δ*3pde* strains. When c-di-GMP-I ON riboswitch Bc2 was transcriptionally fused as a positive control, the β-galactosidase activity of the reporter gene was significantly increased in the three strains (Fig. 3C), indicating that differences in endogenous c-di-GMP concentrations of Δ*2dgc*, BMB171 and Δ*3pde* strains effectively triggered the transcriptional regulation of Bc2. When the test sequence of Bc1 was fused, there was no significant difference in the β-galactosidase activities of the three strains (Fig. 3A and B), and the expression of the reporter gene was always suppressed. After deleting T1 or T2 alone in reporter assays, the β-galactosidase was neither affected in response to different c-di-GMP concentrations (Fig. 3D). This reflects that neither T1 nor T2 seems to respond to intracellular c-di-GMP fluctuations. Moreover, the *in vitro* response of Bc1 to c-di-GMP was also examined, and the similar consequences were obtained. No matter whether T1 or T2 was deleted, no significant change in the pattern of the FL or truncated transcripts was observed with increasing c-di-GMP concentrations (Fig. 2F through H; Fig. S3E). The above experiments appear to imply that Bc1 was unable to change downstream gene expressions in response to different c-di-GMP concentrations.

### Bc1 secondary structure revealed by SHAPE-MaP changed significantly in response to c-di-GMP

**FIG 4:**
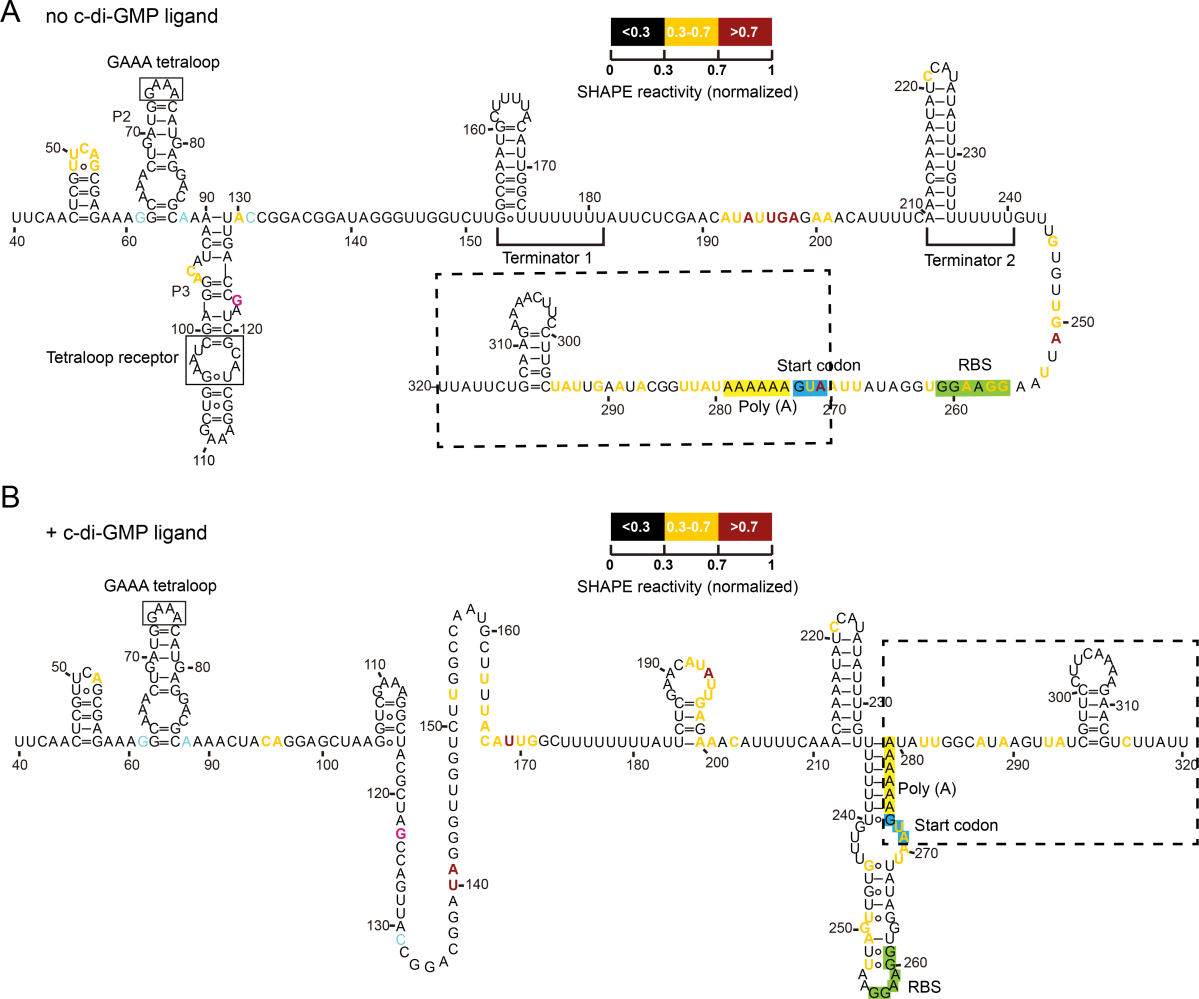
Bc1-*mcpE* transcript undergoes significant conformational changes in response to c-di-GMP identified by SHAPE-MaP. (**A**) In the absence of c-di-GMP, Bc1 forms a dual terminator structure, and the RBS and start codon AUG of the *mcpE* transcript are fully exposed. (**B**) In the presence of c-di-GMP, the dual terminator structure of Bc1 unfolds, and the *mcpE* RBS and start codon AUG are now trapped in the newly formed structure of Bc1. According to the normalized SHAPE reactivity value, each nucleotide in transcript region 40–320 is shown in black (<0.3), rich yellow (0.3–0.7), and rich red (>0.7), respectively. Both the Bc1 tetraloop and tetraloop receptor are indicated by solid black boxes. The P2, P3 stems of Bc1 AD, and terminators T1, T2 are also labeled. The *mcpE* transcript is indicated by a dashed box, with its RBS, start codon AUG, and preceding poly(A) sequence highlighted in green, blue, and yellow, respectively. See also Fig. S4–S6.

To clarify why the downstream genes expression mediated by Bc1 did not change significantly under different c-di-GMP concentrations, we carried out SHAPE-MaP experiments to determine the potential secondary structure of Bc1 upon c-di-GMP addition. The DNA template here encodes the T7 promoter and the entire 5′ UTR up to the first 64 nucleotides of the *mcpE* gene (−2 to +331) (Fig. S4). The secondary structure of the resulting FL transcript was then modeled using SHAPE reactivity as a constraint. Judging from the changes in the reactivity value (Fig. 4; Fig. S5) and Shannon entropy value (Fig. S6) of each base, we found that the conformation of the transcript changed greatly after adding c-di-GMP, especially downstream EP region (Fig. 4).

In the absence of c-di-GMP, the GAAA tetraloop and tetraloop receptor were formed in Bc1 AD (Fig. 4A). The following two typical ρ-independent transcription terminators T1 and T2 appeared, supporting our dual terminator model (Fig. 4A; Fig. S5). Meanwhile, the RBS and start codon AUG of the *mcpE* transcript were fully exposed, which may facilitate translation. A stem-loop structure was formed within the coding sequence (Fig. 4A).

In the presence of c-di-GMP, the conformation of the transcript underwent a dramatic change (Fig. S5 and S6), which was manifested by the unwinding of the stem in AD where the tetraloop receptor was located (Fig. 4B). The T1 terminator became single-stranded. Interestingly, the T2 hairpin structure remained almost intact, with its poly(U) trail fully complementary to the poly(A) string immediately following the *mcpE* start codon AUG (Fig. 4B). We also observed that the *mcpE* RBS and start codon AUG were involved in the stem-loop structure, which might affect the translation and provide the possibility of Bc1’s “transcription-translation” dual regulation. In accordance with the changes in SHAPE reactivity values, residues with reactivity values decreased by more than 0.2 might interact more tightly with c-di-GMP (Fig. S5C), such as A130, U196, G197, A198, A200, A251, A270, A271, A277, and A283 in the transcript corresponding to A79, U146, G147, A148, A150, A201, A220, A221, A227, and U233 of Bc1, respectively. In contrast, residues with increased reactivity values above 0.2 tended to interact more flexibly with c-di-GMP, including G133, U140, A141, U152, 163–171 region (UUUACAUUG), and A192 in the transcript, corresponding to G83, U90, A91, U102, 113–121 region (UUUACAUUG), and A142 of Bc1, respectively. We hypothesized that these residues with great changes in reactivity values might exhibit an important impact on the regulatory mechanism of Bc1.

**FIG 5:**
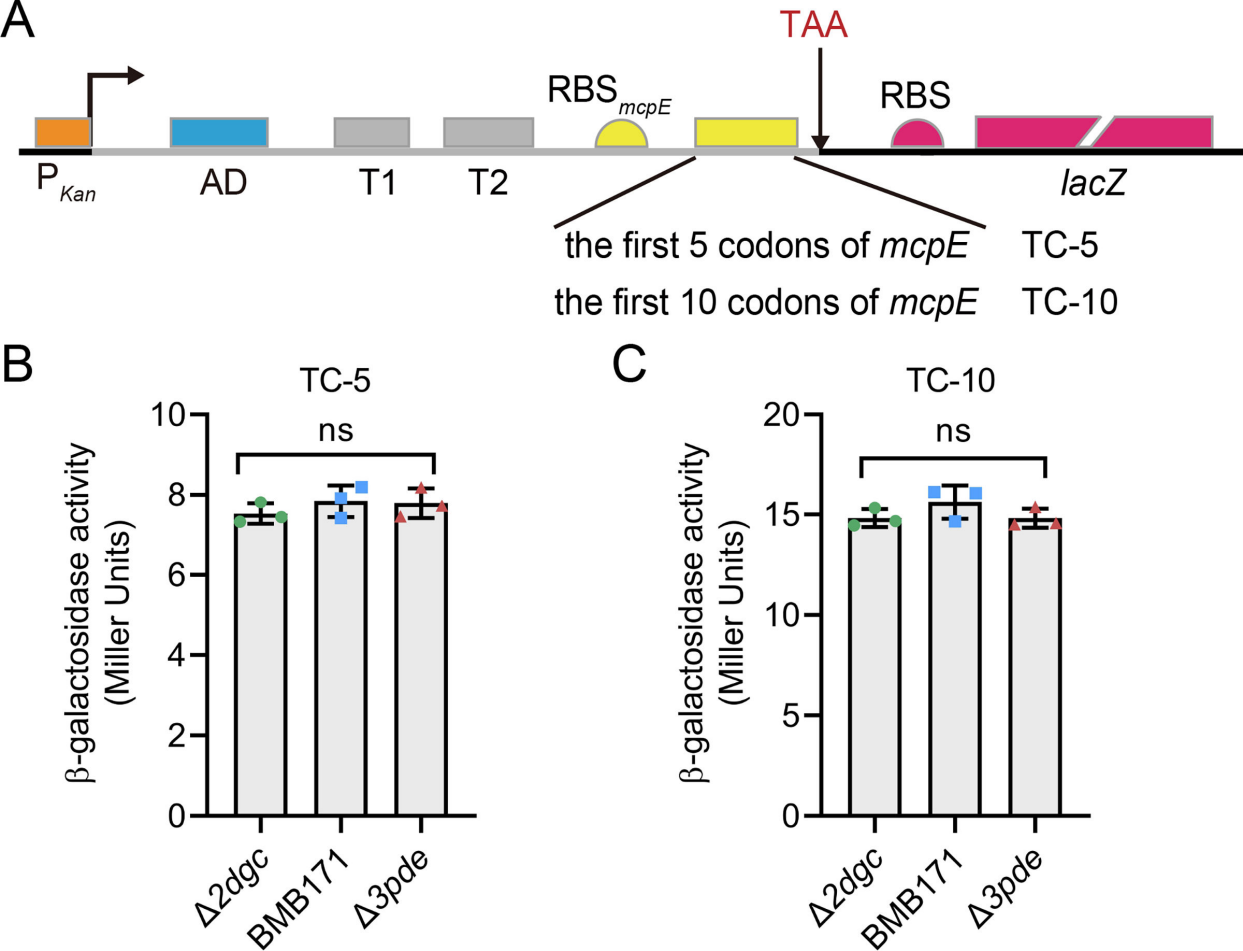
The poly(A) string within preceding *mcpE* coding sequence does not relieve the transcriptional inhibition of Bc1. (**A**) Schematic diagram of transcriptional fusions containing the 5′ UTR coding sequence up to the first 5 or 10 codons of the *mcpE* gene, denoted TC-5 and TC-10, respectively. It is worth noting that a “TAA” (shown in red) is inserted between the coding sequence of the *mcpE* gene and the RBS of the *lacZ* gene in the reporter plasmid. The respective elements are shown as Fig. S3A. (**B**) β-Galactosidase activity assay of Δ*2dgc*, BMB171 and Δ*3pde* strains containing TC-5 transcriptional fusion construct. (**C**) β-Galactosidase activity assay of Δ*2dgc*, BMB171 and Δ*3pde* strains containing TC-10 transcriptional fusion construct. Values in panels *B* and *C* are mean ± standard deviation of three biological replicates, and the significance of the difference was analyzed by one-way ANOVA in GraphPad Prism 8.0.6 with Bonferroni correction. ns means no significance, *P* > 0.05; *P < 0.05; **P < 0.01; ***P < 0.001.

From the SHAPE-MaP data, we focused more on the perfect base pairing between poly(U) in T2 and poly(A) in the preceding *mcpE* transcript (Fig. 4B). We wondered whether this paring could alleviate transcriptional termination of T2, because single-stranded poly(U) is essential for most canonical ρ-independent terminators. To test this hypothesis, we constructed new transcriptional fusion reporter constructs: TC-5 and TC-10, that is, the coding sequence of the 5′ UTR up to the first 5 or 10 codons of the *mcpE* gene, which contains intact poly(A) string, was transcriptionally fused to *lacZ*, respectively (Fig. 5A). Unexpectedly, the β-galactosidase activity of the reporter gene in TC-5 (Fig. 5B) or TC-10 (Fig. 5C) transcriptional fusions was not affected by different c-di-GMP concentrations, demonstrating that the complementary base pairing of poly(U) and poly(A) does not relieve the transcriptional inhibition by T2 and that T2 still plays a major role in repressing the downstream genes. Taken together, SHAPE-MaP confirmed the direct interaction between Bc1 and c-di-GMP *in vitro*, and supported the dual terminator model of Bc1. Notably, even though T1 become single-stranded, its effect might be masked because downstream T2 was always active regardless of c-di-GMP concentrations.

### TF Zur slightly affected the response of Bc1 to c-di-GMP

As certain riboswitches work with other regulatory elements to mediate downstream gene expressions, we conjectured that there may exist some underlying barriers preventing Bc1 from responding to c-di-GMP. To this end, we conducted an in-depth analysis of the adjacent sequences of Bc1. Intriguingly, a 20 nt Fur family TF-binding motif (ATTGAGAAACATTTTCAAAC) was found between the coding sequences of the Bc1 dual terminators (Fig. 1B). This motif is highly conserved among *B. cereus* group bacteria (Fig. S7A). Electrophoretic mobility shift assay (EMSA) results show that Zur (BMB171_RS21350), one of the Fur family TFs in BMB171, directly interacts with the positive control sequence (ttatttttataattGATAATGATAATCATTTATCaatagattgcgtttttc of *dhbA* gene from *B. subtilis*) (Fig. S7B) (72–74). Similarly, we observed that Zur binds directly to the coding sequence of the Bc1 dual terminator (Fig. S7C). The addition of Mn^2+^ did not affect the interaction between Zur and the test sequence *in vitro*, indicating that Zur directly binds to the Bc1 coding sequence independently of Mn^2+^. When the conserved 20 nt motif was mutated, Zur was unable to bind to the mutated probe (Fig. S7D), indicating that the interaction between Zur and the target Bc1 coding sequence is specific.

**FIG 6:**
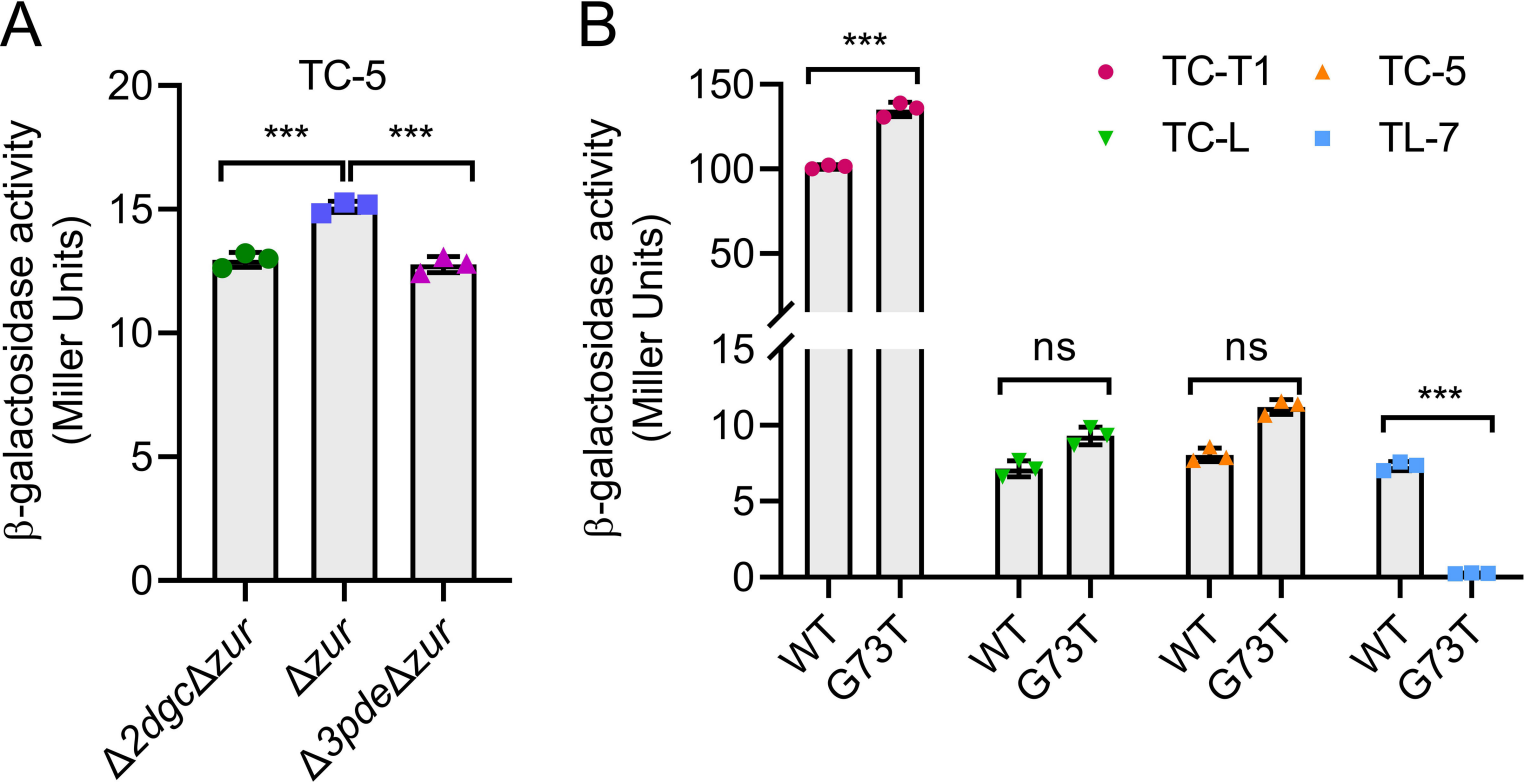
Bc1 has a “transcription-translation” dual regulation mechanism. (**A**) β-Galactosidase activity assay of Δ*2dgc*Δ*zur,* Δ*zur* and Δ*3pde*Δ*zur* strains containing the TC-5 transcriptional fusion construct. (**B**) β-Galactosidase activity assay of BMB171 containing various reporter constructs TC-T1, TC-L, TC-5 and TL-7 upon G73T mutation of Bc1 AD. Values in *A* and *B* are mean ± standard deviation of three biological replicates, and the significance of the difference was analyzed by one-way ANOVA in GraphPad Prism 8.0.6 with Bonferroni correction, ns means no significance, *P* > 0.05; *P < 0.05; **P < 0.01; ***P < 0.001. See also Fig. S7.

The critical region for Zur binding is located between the coding sequences of the Bc1 dual terminators T1 and T2 (Fig. 1B). In light of this, we speculated that their interaction may prevent RNAP from passing through this region (75), thereby repressing transcription of downstream sequences, including downstream EP of Bc1. To confirm this hypothesis, we created Δ*2dgc*Δ*zur*, Δ*zur* and Δ*3pde*Δ*zur* markerless mutants. Surprisingly, the β-galactosidase activities of the Δ*2dgc*Δ*zur* and Δ*3pde*Δ*zur* strains containing the TC-5 transcriptional fusion constructs were significantly reduced compared with Δ*zur* (Fig. 6), suggesting that the presence of Zur indeed affects Bc1’s response to c-di-GMP, even in a minor mode.

## DISCUSSION

### The c-di-GMP riboswitch Bc1 could form an unusual dual terminator

The c-di-GMP-I riboswitch Bc1 from strains of the *B. cereus* group was reported to upregulate the translation of downstream reporter gene in *B. subtilis* (33). Here, we specifically characterized the structure and function of Bc1 in its native genetic context. We found that the long EP of Bc1 had two ρ-independent transcription terminators, T1 and T2 (Fig. 1B). Under normal physiological conditions, T2 was the major contributor to transcriptional inhibition and translational activation, whereas T1 together with AD only had a minor impact on transcription repression (Fig. 2B through D and 5). SHAPE-MaP supported the dual terminator model (Fig. 4). This suggests that poly(U) is not required for T2’s transcriptional regulation, because its hairpin structure remains nearly intact and functional (Fig. 5). The deletion of T1 or T2 alone in *in vitro* transcription termination assays elucidates that T1 and T2 are both effective transcription terminators. However, T1 seemed to be more efficient than T2 *in vitro*, because the most apparent truncated bands were similar in size to TT1 (Fig. 2F through H; Fig. S3E). The discrepancy between the *in vivo* and *in vitro* results may be explained by the fact that the longer TT2 transcript contains an AU-rich region (at T1) and is susceptible to further cleavage by RNase (76), resulting in more abundant TT1. The appearance of shorter transcripts between TT1 and TT2 can demonstrate this (Fig. 2F and G; Fig. S3E). The different performances of T1 and T2 under different conditions also reflect that a variety of intracellular factors may contribute to its physiological functions (77), whereas the *in vivo* conditions were not fully satisfied *in vitro*. Besides, even if the transcripts were denatured in practice, its position on the gels did not exactly match the size indicated by the marker (Fig. 2F through H; Fig. S3E), possibly due to effects such as electrophoresis temperature, gel concentration, and sample amount.

In some cases, dual terminators are more effective than single terminators in transcription repression (78–80). c-di-GMP riboswitches generally control transcription by forming a single ρ-(in)dependent terminator (77, 81), and some do not even contain any terminator (31, 37–39, 68). A riboswitch containing a dual terminator within a single EP is rarely seen in previous studies. The sequence of the Bc1 dual terminator is conserved among bacteria of the *B. cereus* group (Fig. S1). Conclusively, we proposed that under physiological conditions, c-di-GMP-I riboswitch Bc1 could form a “weak-strong” dual terminator and strongly inhibited the transcription of downstream genes.

### Zur mediation provided the possibility for the response of Bc1 to c-di-GMP

The most curious fact of this work is that the expression of genes downstream of Bc1 was not altered by c-di-GMP through a series of *in vivo* assays. Despite repressing transcription with a dual terminator, Bc1 seemed to be locked in an OFF state regardless of c-di-GMP fluctuations. However, after deleting *zur*, the situation was slightly relieved. *In vitro*, the Zur TF (82–87) was found to bind directly to the coding sequence between the Bc1 dual terminators (Fig. S7), which is partly related to the minor effect of Zur on Bc1 (Fig. 6A). Integrating c-di-GMP-I riboswitches with other regulatory elements to regulate the same target genes has become a popular strategy in bacteria (31, 38, 59, 68, 70). For example, in *V. cholerae*, under low or high intracellular c-di-GMP concentrations, the expression of the *tfoY* gene can be promoted through its upstream c-di-GMP-I riboswitch Vc2 or c-di-GMP TF receptor VpsT, respectively (70). Coincidentally, the c-di-GMP-I riboswitch Vc1 promotes the expression of its downstream *gbpA* gene in response to high concentrations of c-di-GMP, whereas at low c-di-GMP concentrations, the cyclic AMP (cAMP)-cAMP receptor protein (CRP) complex can also promote *gbpA* transcription by specifically binding to the *gbpA* promoter region (68). This reminds us that when studying the regulatory mechanism of a given riboswitch, clues might be hidden in its flanking sequences, such as promoter regions, TF-binding motifs, and coding sequences of downstream genes. Test sequences to be fused in reporter assays should be specifically designed, otherwise the actual mechanism of the riboswitches would be obscured.

### Bc1 had an incoherent “transcription-translation” dual regulation mechanism

In this study, no obstacles were found that interfered with Bc1 other than *zur*. However, the most likely structures simulated by SHAPE-MaP contributed to the understanding of Bc1 and c-di-GMP (Fig. 4). Accordingly, we proposed a dual terminator-based “transcription-translation” dual regulation mechanism. Under low c-di-GMP concentrations, Bc1 preferred to forming a dual terminator, hindering the transcription of downstream genes, while the RBS and AUG was fully exposed for ribosomes to initiate translation (Fig. 4A). In line with this, through T1 or T2 deletion analysis, we confirmed that Bc1 downregulated the transcription of its downstream genes, but upregulated its translation (Fig. 3D). T2, instead of T1, was the determinant in opposite dual regulation of Bc1. When c-di-GMP concentration increased, T1 unfolded, but the most surprising finding was that T2 still formed and remained effective in transcriptional inhibition (Fig. 5). The low SHAPE reactivity values (Fig. 4; Fig. S5) of T2, along with its low Shannon entropy values (Fig. S6), confirmed its unique and convincing structures. Owing to this, unwinding of upstream T1 did not overcome the robust regulation mediated by downstream T2 (Fig. 4B and 5). Therefore, no response pattern of Bc1 to c-di-GMP was observed in the background of T2. Besides, it is uncertain whether the conformation of T1 will remain unchanged with increasing c-di-GMP concentration upon T2 deletion. Similar with T2 of Bc1, the SAM-II riboswitch in *Sinorhizobium meliloti* always forms a transcription terminator within EP independent of ligand concentrations (88). Another noticeable change upon c-di-GMP addition was that RBS and the start codon AUG were involved in the formation of a stem-loop structure, which might reduce the ribosome recruitment and cause severe translational inhibition (89, 90). Moreover, translational inhibition could sometimes adversely affect the transcription processes (58). In reporter assays, the G83T mutation in Vc2 disrupts its translational regulation in *Escherichia coli* (33). Similarly, the G73T mutation in Bc1, corresponding to G83T in Vc2 (Fig. S1 and S2), moderately alleviated transcriptional suppression by T1 but fully reinforced the translational inhibition by the Bc1 dual terminator (Fig. 6B). This aligns closely with the impact of T2 deletion (Fig. 3B), supporting the opposite “transcription-translation” dual regulation mechanism of Bc1. Ignoring c-di-GMP addition, the SHAPE-MaP data show the high Shannon entropy values (Fig. S6) in the region of 90–190 except T1, and the region downstream of T2, suggesting the potential existence of alternative structures. Associated with the uncertain mechanism of T2 on translation in the presence of c-di-GMP, we deduced that the dual “transcription-translation” regulation of Bc1 was dynamic when encountering different ligand concentrations, allowing stable expression of downstream genes. Dual regulatory mechanisms have been found in many riboswitches, which normally have long EPs to readily coordinate transcription, translation, or mRNA decay of downstream genes (91–97). Synergistic effect of riboswitches at different levels is what called coherent dual regulation. For instance, the *E. coli ribB* riboswitch represses translation and provokes Rho-dependent transcription termination oppositely (98). The *Legionella pneumophila* guanidine riboswitch upregulates translation of a guanidine efflux pump gene and concomitantly stabilizes the mRNA (88, 99). Additionally, the *E. coli lysC* riboswitch not only inhibits the translation initiation but also exposes RNase E cleavage sites located in the riboswitch EP (92). Meanwhile, there is some incoherent dual regulation by riboswitches. For example, as the intracellular SAM concentration increases, the SAM-II riboswitch in *S. meliloti* enhances the stability of the downstream *metA* gene, but inhibits its translation (88). The exploration of dual regulation riboswitches in the future is expected to garner significant attention, thereby enhancing our comprehension and utilization of RNA regulatory elements.

### There are riboswitches in nature that cannot regulate downstream genes

This research revolved around “poor changes” in downstream gene expression, which could be divided into at least three scenarios: (i) A riboswitch binds to a ligand but its regulation on the downstream gene is counteracted by other elements; (ii) A riboswitch binds to a ligand but it is self-antagonistically regulated; (iii) A riboswitch neither binds to a ligand nor regulates its downstream gene. In general, we tend to assume that a given riboswitch is likely to respond to its specific ligands and regulate downstream genes, especially when its AD is conserved in sequence and distribution. The poor responses of Bc1 under physiological conditions reminded us of recent studies on putative thiamine pyrophosphate (TPP) aptamer in the *Phaeodactylum tricornutum* genome (100). Although the TPP aptamer is highly conserved in more than 20 diatoms genomes, it neither binds nor responds to the ligand TPP, let alone regulates the downstream genes (100). Given the large number and variety of predicted riboswitches, most of them have not yet been experimentally validated (36, 37, 43, 51). Therefore, we could not draw any conclusions about how many riboswitches are highly conserved but do not respond to ligands. This study highlighted that pure bioinformatics and conservative prediction might not be sufficient to infer their true functions. Whether it is a c-di-GMP riboswitch that might be “dormant” in bacteria or a TPP riboswitch in eukaryotes, experiments are indispensable to ensure its effectiveness.

## MATERIALS AND METHODS

### Bacterial strains and growth conditions

The bacterial strains used in this work can be found in Table S3. *B. thuringiensis* BMB171 and its derivatives were grown in lysogeny broth (LB) at 28°C unless otherwise specified. *E. coli* DH5α or BL21(DE3) strains used for cloning were grown in LB broth or LB agar plates at 37°C. When necessary, relevant antibiotics were added to the cultures at the following final concentrations: 25 µg/mL erythromycin, 100 µg/mL ampicillin, 60 U polymyxin. 25 or 50 µg/mL kanamycin (Kan) and 100 µg/mL spectinomycin were used for *E. coli*. 15 µg/mL kanamycin, 0.2%–0.4% D-mannose, and 300 µg/mL spectinomycin were used for *B. thuringiensis*.

### Primers and plasmids construction

The oligonucleotides and plasmids used in this study are listed in Tables S1 and S2 in the Supplemental material.

### RNA isolation, cDNA synthesis, and RT-qPCR

Total RNA was extracted from BMB171 or its derivative strains using TRIzol (AG21101, AG), followed by removal of genomic DNA. The resulting RNA was then reverse-transcribed into cDNA using random primers (11141ES60, Yeasen). RT-qPCR was conducted using SYBR Green dye (11202ES03, Yeasen) and the 2^−ΔΔCT^ method as previously described with modifications (101, 102). Quantitative PCR reactions were carried out in a QuantStudio 3 real-time PCR instrument (Applied Biosystems). The PCR stage conditions were: 95°C for 10 s, 55°C for 30 s, and 72°C for 30 s for 40 cycles. *gapdh* (*BMB171_RS22800*) was used as a reference gene to determine relative transcriptional expression.

### 5′-RACE assay

The 5′-RACE experiment using terminal deoxynucleotidyl transferase (TdT, 2230A, Takara) was performed as previously described with modifications (103). PCR was conducted using primers 5′-RACE adaptor F and Bc1-R in Table S1. The 3′ end of the 5′-RACE adaptor is the oligo (T)_16_ sequence, and the nucleotide after the 5′-RACE adaptor is the TSS.

### Construction of transcriptional and translational fusion strains

Transcriptional and translational fusion constructs were created as previously reported with modifications (103). The target DNA fragment containing the designed pHT1K-*lacZ* homologous fragments on both sides was amplified by PCR, and then inserted into the pHT1K-*lacZ* plasmid digested with *Nco* I-*Bam*H I or *Nco* I-*Sal* I, respectively, to construct transcriptional fusion or translational fusion plasmids (Table S2). The region between the *Bam*H I and *Sal* I site of reporter plasmid pHT1K contains a functional RBS (Fig. S3A), so translation of the *lacZ* reporter gene in transcriptional and translational fusion depends on the native RBS or inserted RBS, respectively. After sequencing (Quintara Biotech Co., Ltd.), the confirmed plasmids were electroporated into the prepared *B. thuringiensis* competent cells to obtain the target strains for β-galactosidase activity assays.

### β-Galactosidase activity assays

β-Galactosidase activity of strains harboring various transcriptional fusion or translational fusion constructs were estimated as previously described (101, 104).

### Markerless gene deletion mediated by homing endonuclease I-*Sce*I

Markerless gene deletion mediated by homing endonuclease I-*Sce*I in *B. thuringiensis* was performed as previously reported (101, 105, 106). The primers, plasmids, and intermediate materials used in this experiment are listed in Tables S1 to S3.

### Markerless gene deletion mediated by CRISPR-Cas9 system

The gene knockout mediated by CRISPR-Cas9 system in *B. thuringiensis* was conducted as previously reported (107, 108) with some changes: First, sgRNA targeting the *zur* gene, obtained from the CHOPCHOP website (http://chopchop.cbu.uib.no/) (109, 110), was used as fragment 1, and the homologous fragments upstream and downstream of the *zur* gene on the BMB171 genome were amplified and overlapped as fragment 2. Fragment 1 and fragment 2 were inserted between the two *Bsa* I and two *Sfi* I restriction sites of the temperature-sensitive vector pJOE8999, respectively, to construct the recombinant vector pJOE8999-sgRNA-UD (*zur*). The recombinant vector was then electroporated into Δ*2dgc*, BMB171, and Δ*3pde* competent cells, and the transformed products were spread on LB plates containing 15 µg/mL kanamycin and 0.2%–0.4% D-mannose for homologous recombination. After cultivation at 37°C for 24 h, the colonies that gradually appeared were picked and cultured in antibiotic-free LB liquid at 37°C. After 5–6 subculturing in antibiotic-free LB liquid, the cultures were isolated by streaking onto the antibiotic-free LB plates at 37°C. Each single colony that did not grow on the kanamycin-containing plate but grew on the plate without antibiotics was picked, and then was verified by PCR and sequencing (Quintara Biotech Co., Ltd.).

### Protein purification and preparation of specific rabbit polyclonal antibodies

The *E. coli* BL21(DE3) strain harboring protein expression plasmids such as pET28a-*mcpE*, pET28b-*gapdhN,* or pET28b-*zur* were grown at 37°C with shaking until OD_600_ reached 0.6, and then isopropyl-β-d-thiogalactoside (IPTG) was added to 0.5 mM. Bacteria were grown at 16°C with shaking (200 r/min) for 14–16 h. Cells were harvested, washed once with binding buffer (20 mM Tris-HCl, 500 mM NaCl, 20 mM imidazole, pH 8.0), resuspended in binding buffer, followed by crushing and centrifugation at 4°C. Supernatants were prepared for the purification of N-terminally 6× His-tagged McpE, and C-terminally 6× His-tagged GapdhN or Zur. Ni-nitrilotriacetic acid (Ni-NTA) affinity purification of these three proteins was performed as previously described (104). After confirmation by SDS-PAGE, purified Zur was used for EMSA. The purified GapdhN and McpE were sent for the preparation of specific rabbit anti-GapdhN polyclonal antibody (Zoonbio Biotech Co., Ltd.) and rabbit anti-McpE polyclonal antibody (AtaGenix Laboratories Co., Ltd.), respectively.

### Electrophoretic mobility shift assays

EMSAs were performed as previously described with some modifications (104, 111). DNA fragments of the Bc1 dual terminator coding sequence and mutant DNA fragments were generated by PCR using Primer 1/Primer 2, and mut-F/mut-R, respectively (Table S1). Positive control check (ck+) DNA fragment of Fur family TFs were prepared using primers CK-F/CK-R (Table S1). Aliquots of 50 µM DNA fragments were added to a total reaction volume of 20 µL containing 100 µM MnCl_2_ and varying amounts of Zur in EMSA buffer (50 mM Tris–HCl, pH 7.5; 10 mM MgCl_2_; 1 mM DTT; 100 mM NaCl). The total mixture was incubated at 25°C for 30 min and then were loaded on a 6% native polyacrylamide gel (30% acrylamide/bis-acrylamide, 2.4 mL; 5× Tris-Borate-EDTA (TBE) buffer, 1.2 mL; 50% glycerol, 0.6 mL; 10% ammonium persulfate, 120 µL; tetramethyl ethylenediamine, 12 µL; H_2_O was added to a total volume of 12 mL). Electrophoresis was conducted at 100 V for 1–1.5 h in 0.5× TBE buffer. Gels were stained in 3× YeaRed (10202ES76, Yeasen, diluted with 0.3 M NaCl) for 20–30 min and finally exposed to UV for imaging.

### Western blot

The Western blot assays were performed as previously reported (102), and GapdhN (BMB171_RS04385, NADP-dependent glyceraldehyde-3-phosphate dehydrogenase) was used as a control loading protein.

### *In vitro* transcription termination assays

*In vitro* transcription was carried out as previously reported with some modifications (103, 112). The DNA template covers the coding sequence from P_m70_ to the first 229 nucleotides of the *mcpE* gene (−60 to +496), or with a T1/T2 deletion. The *in vitro* transcription reaction was as follows: First, 2 U of *E. coli* RNAP holoenzyme (M0551S, New England Biolabs), 2 uL of rNTP mix (N0466S, New England Biolabs, 25 mM of each), and 40 U of recombinant RNase inhibitor (2313A, Takara) were mixed with 200 ng DNA template in 20 µL including 5× *E.coli* RNAP reaction buffer (4 mM Tris–HCl; 20 mM NaCl; 0.02 mM EDTA; 0.2 mM DTT and 10% Glycerol; pH 7.5) (M0551S, New England Biolabs) and incubated at 37°C for 2 h. Then, 15 U of RNase free DNase I (6140, Takara) was added to eliminate the DNA template by incubating at 37°C for 20 min. 2× RNA loading dye (B0363S, New England Biolabs) was added to the mixture or low range ssRNA ladder (N0364S, New England Biolabs), heated at 95°C for 5 min. Denatured samples or ladders were then separated by 5% acrylamide/8 M urea gel electrophoresis at 100 V for 1.5–2 h. The gel was transferred to 0.5× TBE containing 1× SYBR Gold (S11494, Invitrogen) for staining, and finally developed for vision.

### *In vitro* RNA folding and SHAPE probing

The protocol for the SHAPE-MaP method was conducted as previously reported (113, 114). We used the T7-Bc1-F2 and T7-Bc1-R2 primers listed in Table S1 to amplify the DNA template that includes the T7 promoter, the entire 5′ UTR coding sequence up to the first 64 nucleotide of *mcpE* to obtain RNA transcripts according to the manual of the *in vitro* transcription T7 Kit (6140, Takara). RNA transcripts were purified by denaturing polyacrylamide gel electrophoresis and eluted from the gel overnight. After adding an equal volume of DMSO (472301, Sigma-Aldrich) to 5 pmol RNA and 100 pmol c-di-GMP (SML1228, Sigma-Aldrich) (NAI_GMP+) (control group NAI_GMP-), the mixture was incubated in folding buffer [100 mM HEPES (pH 8.0), 100 mM NaCl and 10 mM MgCl_2_] at 37°C for 30 min for refolding. SHAPE reagent 2-methylnicotinic acid imidazolide (NAI, 03–310, Sigma-Aldrich) was added to a final concentration of 10 mM and the mixture was incubated at 37°C for 10 min for RNA modification. Meanwhile, another group of samples (DMSO_GMP+ and DMSO_GMP-) treated with DMSO only were used as no-reagent control. To exclude out specific sequence biases in compound modification, we further added a set of denaturing controls (DC_GMP+ and DC_GMP-). We then denatured the RNA in DC buffer (50 mM pH 8.0 HEPES, 4 mM EDTA and 50% formamide), and modified the RNA with NAI at 95°C. All modified samples were finally purified by RNA affinity columns (RNeasy MinElute; 47014, Qiagen). Purified RNA samples were reverse transcribed using SuperScript II (18064–022, Invitrogen) at 42°C for 3 h. Subsequent library construction was performed according to VAHTS Universal V8 RNA-seq Library Prep Kit for MGI. Finally, we used the MGI platform to perform high-throughput sequencing.

### Quantification and statistical analysis

#### Bioinformatics analysis

Promoters of bacterial genes were predicted using Softberry’s online service (http://www.softberry.com/berry.phtml?topic=bprom&group=programs&subgroup=gfindb) (115). The transcription terminator in mRNA transcript was predicted by Cofold web server (https://www.e-rna.org/cofold/) (116). Sequence alignments were carried out by ClustalW 2.1 web server (https://www.genome.jp/tools-bin/clustalw) (117, 118), and the result was then processed by the ESPript 3.0 web server (https://espript.ibcp.fr/ESPript/cgi-bin/ESPript.cgi) (119). Conservative motifs’ analysis was analyzed by MEME 5.5.2 online service (https://meme-suite.org/meme/tools/meme) (120).

#### Analysis of quantitative data analysis

For RT-qPCR and β-galactosidase activity assays, results are shown as mean ± SD with at least three biological replicates per experiment, which are also indicated in the figure legends. Statistical significance was analyzed by one-way analysis of variance (ANOVA) or two-way ANOVA with Bonferroni correction in GraphPad Prism 8.0.6. A *P* value of <0.05 was considered statistically significant, and a *P* value of >0.05 was considered statistically non-significant (ns).

#### SHAPE-MaP data analysis

We used RNA Framework v2.8.2 (https://github.com/dincarnato/RNAFramework) for SHAPE data processing (121, 122). Among them, the rf-map module was used to compare with the reference genome (parameters: -ctn -cmn 0 -cqo -cq5 20 -b2); the rf-count module was used to calculate the mutation rate of each base (parameters: -m -rd); and the rf-norm module was used to standardize the data of the experimental group and the control group (parameters: -sm 3 nm 3 mm 1). To simulate the secondary structure of the Bc1-containing RNA, we used the rf-fold module and the ViennaRNA package v2.4.14 (parameters: -ct -w -fw 100 -fo 10 wt 30 -pw 100 -po 10 -dp -sh -nlp - md 300), and used SHAPE data as soft constraints to predict the RNA folding structure in three steps. In the first step, a 100 nt window was slid along the RNA sequence, offset by 10 nt along the folded window and the assignment function was calculated. In the second step, the base pairing rates of all windows were averaged, and base pairs with a probability >99% were retained as hard constraints in the third step; in addition, the Shannon entropy of each base was also calculated by the base pairing probability. In the third step, a 100 nt window was slid along the RNA sequence to perform minimum free energy (MFE) folding with the SHAPE probe data as a soft constraint and base pairs occurring in >50% of the window were retained to obtain the final secondary structure of riboswitch.

## Data Availability

All data reported in this paper will be shared by the lead contact upon request. This paper does not report original code. Any additional information required to reanalyze the data reported in this work paper is available from the lead contact upon request. Plasmids generated in this study will be available upon request with a completed Materials Transfer Agreement. This study did not generate new unique reagents.

## SUPPLEMENTAL MATERIAL

Supplemental tables and figures spectrum.00450-24-s0001.docx Tables S1-S3; Fig. S1-S7.

ASM does not own the copyrights to Supplemental Material that may be linked to, or accessed through, an article. The authors have granted ASM a non-exclusive, world-wide license to publish the Supplemental Material files. Please contact the corresponding author directly for reuse.
