# Supplementary material for: Characterization of the dual regulation by a c-di-GMP riboswitch Bc1 with a long expression platform from Bacillus thuringiensis
Source: Microbiol Spectr. 2024 May 31;12(7):e00450-24. doi: 10.1128/spectrum.00450-24 (PMC11218506; doi:10.1128/spectrum.00450-24)
Supplement: Supplemental tables and figures — Tables S1-S3; Fig. S1-S7. [file spectrum.00450-24-s0001.docx]

# Supplementary files *for*

**Characterization of the dual regulation by a c-di-GMP riboswitch Bc1 with a long expression platform from *Bacillus thuringiensis***

Lu Liu, Dehua Luo, Yongji Zhang, Dingqi Liu, Kang Yin, Qing Tang, Shan-Ho Chou, Jin He*

National Key Laboratory of Agricultural Microbiology & Hubei Hongshan Laboratory, College of Life Science and Technology, Huazhong Agricultural University

### Supplementary Table S1. Oligonucleotides used in this study.

| **Primer name** | **Sequence (5'→3')** | **Characteristics** |
| --- | --- | --- |
| **5'-RACE assay** | | |
| 5'-RACE adaptor-F | GACCACGCGTATCGATGTCGACTTTTTTTTTTTTTTTT | Forward primer |
| Bc1-R | CTCCTGTTTCAATAGACTGCCTTTCAAATAAAGACATC | Reverse primer |
| **Gene knockout mediated by I-*Sce*I** | |  |
| U-Bc1-S-F | CGGAGACCTGTACACCCGGGTGTTGAGAAGCGATT | Forward primer |
| U-Bc1-S-R | TCTCAATATGTTCGAAACGAGTTGAACGATATGGGAG | Reverse primer |
| D-Bc1-S-F | CTCCCATATCGTTCAACTCGTTTCGAACATATTGAG | Forward primer |
| D-Bc1-S-R | CCATGCATGTCGACGGATCCGAGTAAACCCTGTAT | Reverse primer |
| U-Bc1-L-F | GCGGCCGGAGACCTGTACACCCGGGAGATAGTCCATTCCTCCAGC | Forward primer |
| U-Bc1-L-R | ATTAATATCCACCTTCCTTACGCGTAACGAGTTGAACGATATGGGAG | Reverse primer |
| D-Bc1-L-F | CCCATATCGTTCAACTCGTTACGCGTAAGGAAGGTGGATATTAATG | Forward primer |
| D-Bc1-L-R | TGGTACCATGCATGTCGACGGATCCTTCATCTTCATATACTTCTG | Reverse primer |
| pRP1028-UF | CGTCTTACTGTCGGGAATTGATGCAG | Forward primer |
| pRP1028-DR | GTGCGAATAAGGGACAGTGAAGAAGG | Reverse primer |
| gU-UBc1-F | GCAGCTATCATACAAGCGATGAAAGT | Forward primer |
| U-Bc1-F | CCCGAAGAAATCCTTGTTATTTATAC | Forward primer |
| D-Bc1-R | CCAGTTTTTTCAATACTATTTCTCCTG | Reverse primer |
| **Genome editing mediated by CRISPR-Cas9 system** | | |
| *zur*-sg-F | AATGTACCTACGCGAGACCatatgaaagacgattatccaGGTCTCAGTTTTAGAGCTAG | Forward primer |
| *zur*-sg-R | CTAGCTCTAAAACTGAGACCtggataatcgtctttcatatGGTCTCGCGTAGGTACATT | Reverse primer |
| U-z*ur*-F (*Sfi* I) | AGGCCAACGAGGCCacttgcaaatggatttaacac | Forward primer |
| U-z*ur*-R (cas) | cagttacataaccgacgagcttacctatccctactttatgcag | Reverse primer |
| D-z*ur*-F (cas) | ctgcataaagtagggataggtaagctcgtcggttatgtaactg | Forward primer |
| D-z*ur*-R (*Sfi* I) | AGGCCTTATTGGCCaaatacactacgtatttcattaag | Reverse primer |
| sg-F | GCGTGTGATGCGAATTCTTGACC | Forward primer |
| sg-R | AAAAGCACCGACTCGGTGCCAC | Reverse primer |
| D-D-R (8999) | GAGTCAGCTAGGAGGTGACTGAAG | Reverse primer |
| *zur*-F | ggtacaattgttatgatagcagttc | Forward primer |
| *zur*-R | gaaggttgccacaatatttgtgagc | Reverse primer |
| **Protein expression** | | |
| *mcpE*-F (*Bam*H I) | CGCGGATCCATGAAAAAATATTGGCATAAGTTAT | Forward primer |
| *mcpE*-R (*Hind* III) | CCCAAGCTTTTATTCGCTTTTAAACTCACCAATA | Reverse primer |
| *gapN*-F (*Nco I*) | actttaagaaggagatataccatggcaatgacaactagcaatacgtac | Forward primer |
| *gapN*-R (*Xho* I) | gtggtggtggtggtggtgctcgagaactaagtttaatacagttac | Reverse primer |
| *zur*-F(28b) | actttaagaaggagatataccatggCAatgaatctaacagaagctttac | Forward primer |
| *zur*-R(28b) | agtggtggtggtggtggtgctcgagctttgcacattttggacaacgacc | Reverse primer |
| T7-F | TAATACGACTCACTATAGGG | Forward primer |
| T7-R | GCTAGTTATTGCTCAGCGG | Reverse primer |
| **EMSA** | | |
| Primer 1 | Ccaatgcttttacattggcttttttttattctcgaacatattgagaaacattttcaaac | Forward primer |
| Primer 2 | gtttgaaaatgtttctcaatatgttcgagaataaaaaaaagccaatgtaaaagcattgG | Reverse primer |
| CK-F | TTATTTTTATAATTGATAATGATAATCATTATCAATAGATTGCGTTTTTC | Forward primer |
| CK-R | GAAAAACGCAATCTATTGATAATGATTATCATTATCAATTATAAAAATAA | Reverse primer |
| Mut-F | ccaatgcttttacattggcttttttttattctcgaacatGCCACACCCATAAGCAGCCT | Forward primer |
| Mut-R | AGGCTGCTTATGGGTGTGGCatgttcgagaataaaaaaaagccaatgtaaaagcattgg | Reverse primer |
| **RT-qPCR assay** | | |
| Q*gapdh*-F | GCGCCTGTTGTGAAGGTGTT | Forward primer |
| Q*gapdh*-R | GCGCTTTCGCAGCACCTGTCGTT | Reverse primer |
| QBc1-F | GCGAGAAAGGCAAACTGATGG | Forward primer |
| QBc1-R | CATTGGCCAAGACCAACCCT | Reverse primer |
| Q*mcpE*-F | TGAACAACCCGCTCATTGGA | Forward primer |
| Q*mcpE*-R | AGCGAGTGACGCACGATAAA | Reverse primer |
| **Construction of transcriptional and translational fusion plasmids** | | |
| pHT1K-F(SC) | GATCACTATAGGGCCATGGGGATCCCCAGCTTGTTGATAC | Forward primer |
| pHT1K-R(SC) | TATCAACAAGCTGGGGATCCCCATGGCCCTATAGTGATCG | Reverse primer |
| P*_Kan_*-F (T1K) | CGATCACTATAGGGCCATGGCATTTGAGGTGATAGGTAAGATTATACCGAGG | Forward primer |
| P*_Kan_*-R (T1K) | TATCAACAAGCTGGGGATCCCCCCAAGAAGCTAATTATAACAAGACGAAC | Reverse primer |
| UTR-F (T1K) | CGATCACTATAGGGCCATGGAGAGAATAAATTAATTCTCATAGAACTCCCATATCG | Forward primer |
| P*_Kan_*-R (UTR) | CTATGAGAATTAATTTATTCTCTCCCCAAGAAGCTAATTATAACAAGACGAAC | Reverse primer |
| UTR-F (P*_Kan_*) | ATAATTAGCTTCTTGGGGAGAGAATAAATTAATTCTCATAGAACTCCCATATCG | Forward primer |
| UTR-R (TC-T1) | TATCAACAAGCTGGGGATCCGAATAAAAAAAAGCC | Reverse primer |
| UTR-R (TC-L) | TATCAACAAGCTGGGGATCCTTATAATATCCACCTTCCTTAATC | Reverse primer |
| UTR-R (TC-S) | TATCAACAAGCTGGGGATCCAATCAACACAAAC | Reverse primer |
| UTR-R (TL-2) | CCAGGGTTTTCCCGGTCGACTTTCATTAATATCCACCTTCCTTAATC | Reverse primer |
| UTR-R (TL-7) | CCAGGGTTTTCCCGGTCGACCTTATGCCAATATTTTTTC | Reverse primer |
| UTR-R (TL-10) | CCAGGGTTTTCCCGGTCGACGAACGATAACTTATGCC | Reverse primer |
| UTR-R (TL-20) | CCAGGGTTTTCCCGGTCGACTAATACGGTTAATAAGACG | Reverse primer |
| P*_Kan_*-R (Bc2) | GGACGGTTATAATTCAATACTCCCCCAAGAAGCTAATTATAAC | Reverse primer |
| UTR-F (P*_Kan_*)Bc2 | GTTATAATTAGCTTCTTGGGGGAGTATTGAATTATAACCG | Forward primer |
| UTR-R (Bc2) | GTATCAACAAGCTGGGGATCCATTTTTTATATTTATATAAC | Reverse primer |
| ΔT1-F | cggatagggttggtcttattctcgaacatattgag | Forward primer |
| ΔT1-R | ctcaatatgttcgagaataagaccaaccctatccg | Reverse primer |
| ΔT2-F | catattgagaaacattttcgtttgtgttgattaaggaag | Forward primer |
| ΔT2-R | cttccttaatcaacacaaacgaaaatgtttctcaatatg | Reverse primer |
| UTR-R (TC-5) | GTATCAACAAGCTGGGGATCCTTACCAATATTTTTTCATTAATATCC | Reverse primer |
| UTR-R (TC-10) | GTATCAACAAGCTGGGGATCCTTAGAACGATAACTTATGCC | Reverse primer |
| ***in vitro* transcription termination assays** | | |
| Pm70-F | CGATCACTATAGGGCCATGGaatttgtatttatatctct | Forward primer |
| J23119-Bc1-F | GATCACttgacagctagctcagtcctaggtataatactagtGaaagagaataaattaat | Forward primer |
| Bc1-R2 | ttcactttttcggctgtaggatctttcgccatgtctc | Reverse primer |
| **SHAPE-MaP analysis** | | |
| T7-Bc1-F2 | GATCACTAATACGACTCACTATAGGGAAAGAGAATAAATTAATTC | Forward primer |
| T7-Bc1-R2 | TAACTAATACGGTTAATAAGACGTTC | Reverse primer |

### Supplementary Table S2. Plasmids used in this study.

| **Plasmid name** | **Characteristics** | **Origins** |
| --- | --- | --- |
| pMD19T | a universally commercial cloning vector | Takara |
| pMD19T-5'-RACE-*mcpE* | for identification of TSS of *mcpE* in 5'-RACE | This study |
| pMD19T-T7-Bc1 | A DNA template containing a T7 promoter, 5' UTR coding sequence and the first 64 nucleotides of *mcpE* gene was integrated into pMD19T | This study |
| pRP1028 | *B. thuringiensis*-*E. coli* shuttle plasmid, temperature sensitive, containing *turbo*-*rfp* gene and an I-*Sce*I recognition and cleavage site | (1) |
| pRP1028-Bc1-S (UD) | contains upstream and downstream homologous fragments of Bc1-S | This study |
| pRP1028-Bc1-L (UD) | contains upstream and downstream homologous fragments of Bc1-L | This study |
| pSS1827 | *B. thuringiensis*-*E. coli* shuttle helper plasmid for conjugative transfer | (1) |
| pSS4332 | *B. thuringiensis*-*E. coli* shuttle plasmid, encode I-*Sce*I homing endonuclease and GFP | (1) |
| pJOE8999 | *B. subtilis -E. coli* shuttle plasmid carrying a *cas9* gene under the control of mannose-inducible promoter P*_manP_*, Kan^R^, for genome editing of *Bacillus* | (2, 3) |
| pJOE8999-sg-z*ur* | pJOE8999 with a specific sgRNA targeting to *zur* (*BMB171_RS21350*), an intermediate plasmid | This work |
| pJOE8999-sg-z*ur* (UD) | pJOE8999-sg-*zur* with the upstream and downstream fragment of z*ur* | This work |
| pET28a | a commercial vector for hexahistidine fusion | Novagen |
| pET28a-*mcpE* | for 6× His-tagged McpE expression | This study |
| pET28b | a commercial vector for hexahistidine fusion | Novagen |
| pET28b-*gapdhN* | for fused GapdhN-6 × His expression | This study |
| pET28b-*zur* | for fused Zur-6 × His expression | This study |
| pHT1K | *B. thuringiensis*-*E. coli* shuttle plasmid, carrying a *lacZ* reporter gene with an RBS | Lab stock |
| pHT1K-Pnull-*lacZ* | pHT1K-*lacZ* carries a null promoter | This study |
| pHT1K-P*_Kan_*-*lacZ* | a heterogenous P*_Kan_* constitutive promoter from *Bacillus subtilis* Bs168 was inserted into pHT1K-*lacZ* | This study |
| pHT1K-5' UTR-*lacZ* (TC) | the coding sequence of *mcpE* 5' UTR was inserted into a promoterless pHT1K-*lacZ* | This study |
| pHT1K-5' UTR-*lacZ* (TL) | the coding sequence of 5' UTR and the first 10 codons of *mcpE* was inserted into a promoterless pHT1K-*lacZ* | This study |
| pHT1K-P*_Kan_*-5' UTR-*lacZ* (TC-T1) | the coding sequence of *mcpE* 5' UTR whose 3'-end ends behind the poly (T) of T1 was fused with *lacZ* transcriptionally | This study |
| pHT1K-P*_Kan_*-5' UTR-*lacZ* (TC-S) | the coding sequence of *mcpE* 5' UTR whose 3'-end ends before SD sequence was fused with *lacZ* transcriptionally | This study |
| pHT1K-P*_Kan_*-5' UTR-*lacZ* (TC-L) | the coding sequence of *mcpE* 5' UTR and an extra TAA was fused with *lacZ* transcriptionally | This study |
| pHT1K-P*_Kan_*-5' UTR-*lacZ* (TC-5) | the coding sequence of 5' UTR and the first 5 codons of *mcpE*, as well as an extra TAA was fused with *lacZ* transcriptionally | This study |
| pHT1K-P*_Kan_*-ΔT1-*lacZ* (TC-5_ΔT1_) | the coding sequence of 5' UTR and the first 5 codons of *mcpE* upon T1 deletion, as well as an extra TAA was fused with *lacZ* transcriptionally | This study |
| pHT1K-P*_Kan_*-ΔT2-*lacZ* (TC-5_ΔT2_) | the coding sequence of 5' UTR and the first 5 codons of *mcpE* upon T2 deletion, as well as an extra TAA was fused with *lacZ* transcriptionally | This study |
| pHT1K-P*_Kan_*-5' UTR-*lacZ* (TC-10) | the coding sequence of 5' UTR and the first 10 codons of *mcpE*, as well as an extra TAA stop codon was fused with *lacZ* transcriptionally | This study |
| pHT1K-P*_Kan_*-5' UTR-*lacZ* (TL-2) | the coding sequence of 5' UTR and first 2 codons of *mcpE* was fused with *lacZ* translationally | This study |
| pHT1K-P*_Kan_*-5' UTR-*lacZ* (TL-7) | the coding sequence of 5' UTR and first 7 codons of *mcpE* was fused with *lacZ* translationally | This study |
| pHT1K-P*_Kan_*-ΔT1-*lacZ* (TL-7_ΔT1_) | the coding sequence of 5' UTR and first 7 codons of *mcpE* upon T1 alone deletion was fused with *lacZ* translationally | This study |
| pHT1K-P*_Kan_*-ΔT2-*lacZ* (TL-7_ΔT2_) | the coding sequence of 5' UTR and first 7 codons of *mcpE* upon T2 alone deletion was fused with *lacZ* translationally | This study |
| pHT1K-P*_Kan_*-5' UTR-*lacZ* (TL-10) | the coding sequence of 5' UTR and first 10 codons of *mcpE* was fused with *lacZ* translationally | This study |
| pHT1K-P*_Kan_*-5' UTR-*lacZ* (TL-20) | the coding sequence of 5' UTR and first 20 codons of *mcpE* was fused with *lacZ* translationally | This study |
| pHT1K-P*_Kan_*-5' UTR-*lacZ* (TC-Bc2) | the coding sequence of 5' UTR of Bc2*-cap* was fused with *lacZ* transcriptionally | This study |

### Supplementary Table S3. Strains used in this study.

| **Strains** | **Characteristics** | **Origins** |
| --- | --- | --- |
| BMB171 | an acrystalliferous mutant of wild type strain YBT-1463, which is subsp. kurstaki and a highly toxic wild strain to Lepidoptera | (4) |
| Δ*2dgc* | In frame deletion of *cdgA (BMB171_RS27040)* and *cdgB* (*BMB171_RS20080*) of BMB171 | (5, 6) |
| Δ*3pde* | In frame deletion of *cdgH* (*BMB171_RS02850*), *cdgF* (*BMB171_RS03240*), and *cdgE (BMB171_RS18570)* of BMB171 | (5, 6) |
| ΔBc1-S | In frame deletion of Bc1-S of BMB171 | This study |
| ΔBc1-L | In frame deletion of Bc1-L of BMB171 | This study |
| DH5α/pJOE8999 | DH5α carrying pJOE8999 | This study |
| DH5α/pJOE8999-sg-z*ur* | DH5α carrying pJOE8999-sg-z*ur* | This study |
| DH5α/pJOE8999-sg-z*ur* (UD) | DH5α carrying pJOE8999-sg-*zur* (UD) | This study |
| Δ*2dgc*Δ*zur* | In frame deletion of *zur* (*BMB171_RS21350*) of Δ*2dgc* | This study |
| Δ*zur* | In frame deletion of *zur* gene of BMB171 | This study |
| Δ*3pde*Δ*zur* | In frame deletion of *zur* gene of Δ*3pde* | This study |
| DH5α | *E. coli* B; F^-^ *recA* *lacZ*M15 | Lab stock |
| BL21(DE3) | *E. coli* B; F^-^*ompT* r_B_^-^ m_B_^-^ (λDE3) | Lab stock |
| DH5α/pMD19T-5'-RACE-*mcpE* | DH5α carrying a pMD19T-5'-RACE-*mcpE* plasmid for 5'-RACE | This study |
| DH5α/pMD19T-T7-Bc1 | DH5α carrying a pMD19T-T7-Bc1 plasmid for DNA template in SHAPE-MaP | This study |
| BL21(DE3)/pET28a-*mcpE* | *E. coli* B; F^-^*ompT* r_B_^-^ m_B_^-^ (λDE3); a strain used for McpE induction and expression | This study |
| BL21(DE3)/pET28b-z*ur* | BL21(DE3) carrying the pET28b-z*ur* | This study |
| Δ*2dgc*/Pnull | Δ*2dgc* carrying the pHT1K-Pnull-*lacZ* | This study |
| BMB171/Pnull | BMB171 carrying the pHT1K-Pnull-*lacZ* | This study |
| Δ*3pde*/Pnull | Δ3pde carrying the pHT1K-Pnull-*lacZ* | This study |
| Δ*2dgc*/P*_Kan_* | Δ*2dgc* carrying the pHT1K-P*_Kan_*-*lacZ* | This study |
| BMB171/P*_Kan_* | BMB171 carrying the pHT1K-P*_Kan_*-*lacZ* | This study |
| Δ*3pde*/P*_Kan_* | Δ*3pde* carrying the pHT1K-P*_Kan_*-*lacZ* | This study |
| Δ*2dgc*/5' UTR (TC) | Δ*2dgc* carrying the pHT1K-5' UTR-*lacZ* (TC) | This study |
| BMB171/5' UTR (TC) | BMB171 carrying the pHT1K-5' UTR-*lacZ* (TC) | This study |
| Δ*3pde*/5' UTR (TC) | Δ*3pde* carrying the pHT1K-5' UTR-*lacZ* (TC) | This study |
| Δ*2dgc*/5' UTR (TL) | Δ*2dgc* carrying the pHT1K-5' UTR-*lacZ* (TL) | This study |
| BMB171/5' UTR (TL) | BMB171 carrying the pHT1K-5' UTR-*lacZ* (TL) | This study |
| Δ*3pde*/5' UTR (TL) | Δ*3pde* carrying the pHT1K-5' UTR-*lacZ* (TL) | This study |
| Δ*2dgc*/TC-T1 | Δ*2dgc* carrying the pHT1K-P*_Kan_*-5' UTR-*lacZ* (TC-T1) | This study |
| BMB171/TC-T1 | BMB171 carrying the pHT1K-P*_Kan_*-5' UTR-*lacZ* (TC-T1) | This study |
| Δ*3pde*/TC-T1 | Δ*3pde* carrying the pHT1K-P*_Kan_*-5' UTR-*lacZ* (TC-T1) | This study |
| BMB171/TC-S | BMB171 carrying the pHT1K-P*_Kan_*-5' UTR-*lacZ* (TC-S) | This study |
| Δ*2dgc*/TC-L | Δ*2dgc* carrying the pHT1K-P*_Kan_*-5' UTR-*lacZ* (TC-L) | This study |
| BMB171/TC-L | BMB171 carrying the pHT1K-P*_Kan_*-5' UTR-*lacZ* (TC-L) | This study |
| Δ*3pde*/TC-L | Δ*3pde* carrying the pHT1K-P*_Kan_*-5' UTR-*lacZ* (TC-L) | This study |
| BMB171/TL-2 | BMB171 carrying the pHT1K-P*_Kan_*-5' UTR-*lacZ* (TL-2) | This study |
| Δ*2dgc*/TL-7 | Δ*2dgc* carrying the pHT1K-P*_Kan_*-5' UTR-*lacZ* (TL-7) | This study |
| BMB171/TL-7 | BMB171 carrying the pHT1K-P*_Kan_*-5' UTR-*lacZ* (TL-7) | This study |
| Δ*3pde*/TL-7 | Δ*3pde* carrying the pHT1K-P*_Kan_*-5' UTR-*lacZ* (TL-7) | This study |
| Δ*2dgc*/TL-7_ΔT1_ | Δ*2dgc* carrying the pHT1K-P*_Kan_*-ΔT1-*lacZ* (TL-7_ΔT1_) | This study |
| BMB171/TL-7_ΔT1_ | BMB171 carrying the pHT1K-P*_Kan_*-ΔT1-*lacZ* (TL-7_ΔT1_) | This study |
| Δ*3pde*/TL-7_ΔT1_ | Δ*3pde* carrying the pHT1K-P*_Kan_*-ΔT1-*lacZ* (TL-7_ΔT1_) | This study |
| Δ*2dgc*/TL-7_ΔT2_ | Δ*2dgc* carrying the pHT1K-P*_Kan_*-ΔT2-*lacZ* (TL-7_ΔT2_) | This study |
| BMB171/TL-7_ΔT2_ | BMB171 carrying the pHT1K-P*_Kan_*-ΔT2-*lacZ* (TL-7_ΔT2_) | This study |
| Δ*3pde*/TL-7_ΔT2_ | Δ*3pde* carrying the pHT1K-P*_Kan_*-ΔT2-*lacZ* (TL-7_ΔT2_) | This study |
| BMB171/TL-10 | BMB171 carrying the pHT1K-P*_Kan_*-5' UTR-*lacZ* (TL-10) | This study |
| BMB171/TL-20 | BMB171 carrying the pHT1K-P*_Kan_*-5' UTR-*lacZ* (TL-20) | This study |
| Δ*2dgc*/TC-5 | Δ*2dgc* carrying the pHT1K-P*_Kan_*-5' UTR-*lacZ* (TC-5) | This study |
| BMB171/TC-5 | BMB171 carrying the pHT1K-P*_Kan_*-5' UTR-*lacZ* (TC-5) | This study |
| Δ*3pde*/TC-5 | Δ*3pde* carrying the pHT1K-P*_Kan_*-5' UTR-*lacZ* (TC-5) | This study |
| Δ*2dgc*/TC-5_ΔT1_ | Δ*2dgc* carrying the pHT1K-P*_Kan_*-ΔT1-*lacZ* (TC-5_ΔT1_) | This study |
| BMB171/TC-5_ΔT1_ | BMB171 carrying the pHT1K-P*_Kan_*-ΔT1-*lacZ* (TC-5_ΔT1_) | This study |
| Δ*3pde*/TC-5_ΔT1_ | Δ*3pde* carrying the pHT1K-P*_Kan_*-ΔT1-*lacZ* (TC-5_ΔT1_) | This study |
| Δ*2dgc*/TC-5_ΔT2_ | Δ*2dgc* carrying the pHT1K-P*_Kan_*-ΔT2-*lacZ* (TC-5_ΔT2_) | This study |
| BMB171/TC-5_ΔT2_ | BMB171 carrying the pHT1K-P*_Kan_*-ΔT2-*lacZ* (TC-5_ΔT2_) | This study |
| Δ*3pde*/TC-5_ΔT2_ | Δ*3pde* carrying the pHT1K-P*_Kan_*-ΔT2-*lacZ* (TC-5_ΔT2_) | This study |
| Δ*2dgc*/TC-10 | Δ*2dgc* carrying the pHT1K-P*_Kan_*-5' UTR-*lacZ* (TC-10) | This study |
| BMB171/TC-10 | BMB171 carrying the pHT1K-P*_Kan_*-5' UTR-*lacZ* (TC-10) | This study |
| Δ*3pde*/TC-10 | Δ*3pde* carrying the pHT1K-P*_Kan_*-5' UTR-*lacZ* (TC-10) | This study |

**
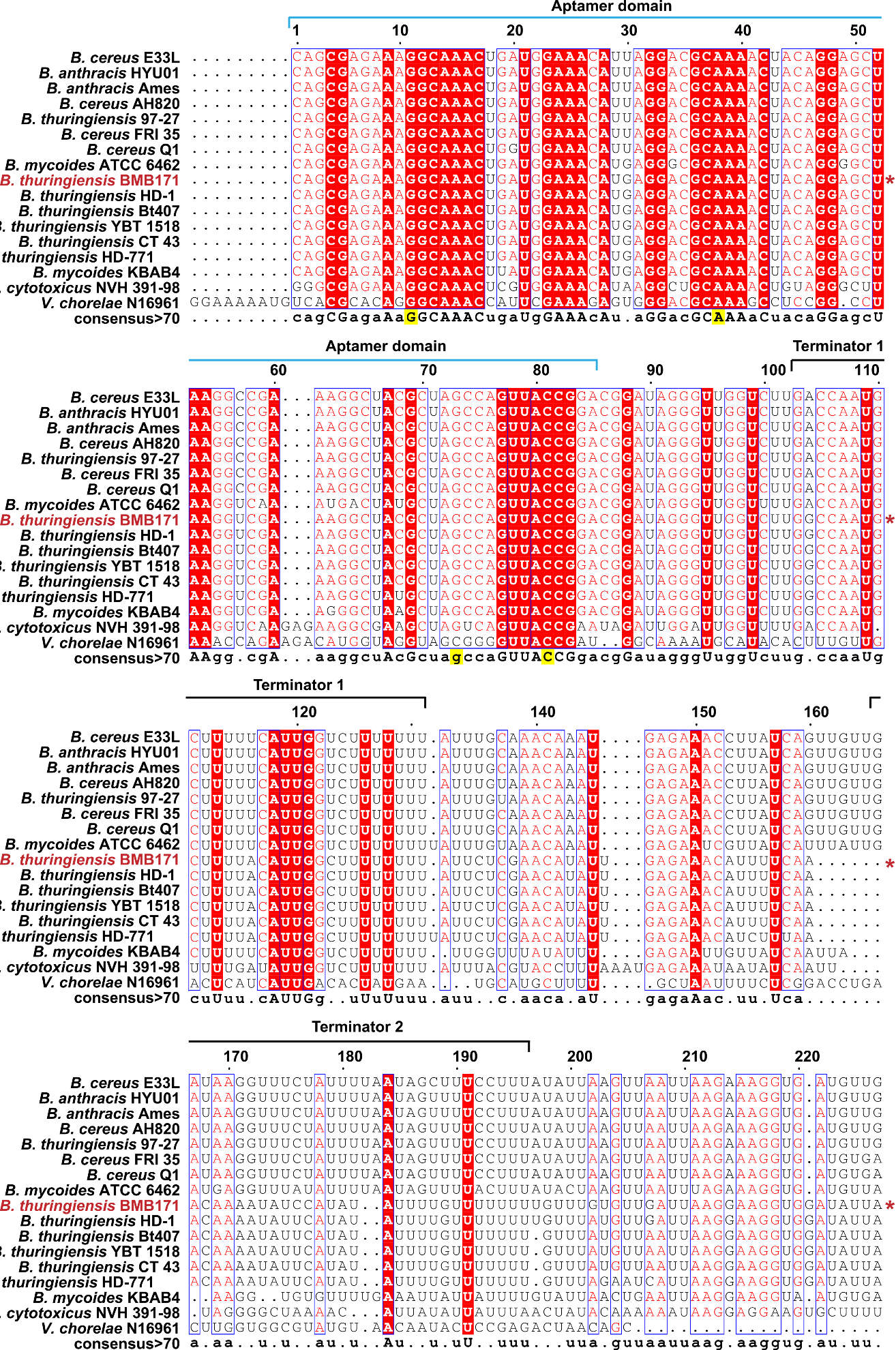
**

**Supplementary Figure S1. Compared with the classical c-di-GMP riboswitch Vc2 from the *V. cholerae* O1 El Tor N16961 strain, Bc1 from 16 *B. cereus* group strains is conserved, related to Fig. 1.** Bc1 from the BMB171 strain in this study is highlighted in red with an asterisk on the right. The aptamer domain, Terminator 1, and Terminator 2 of Bc1 are indicated as blue and black lines, respectively. The red background indicates the 16 fully conserved nucleotides of the Bc1 riboswitches, highlighted in capital letters, and nucleotides with >70% conservation are indicated by lowercase letters. G11, A38, G73, and C81 of Bc1 correspond to the crucial nucleotides G20, A47, G83, and C92 in Vc2, respectively, and are highlighted in yellow.


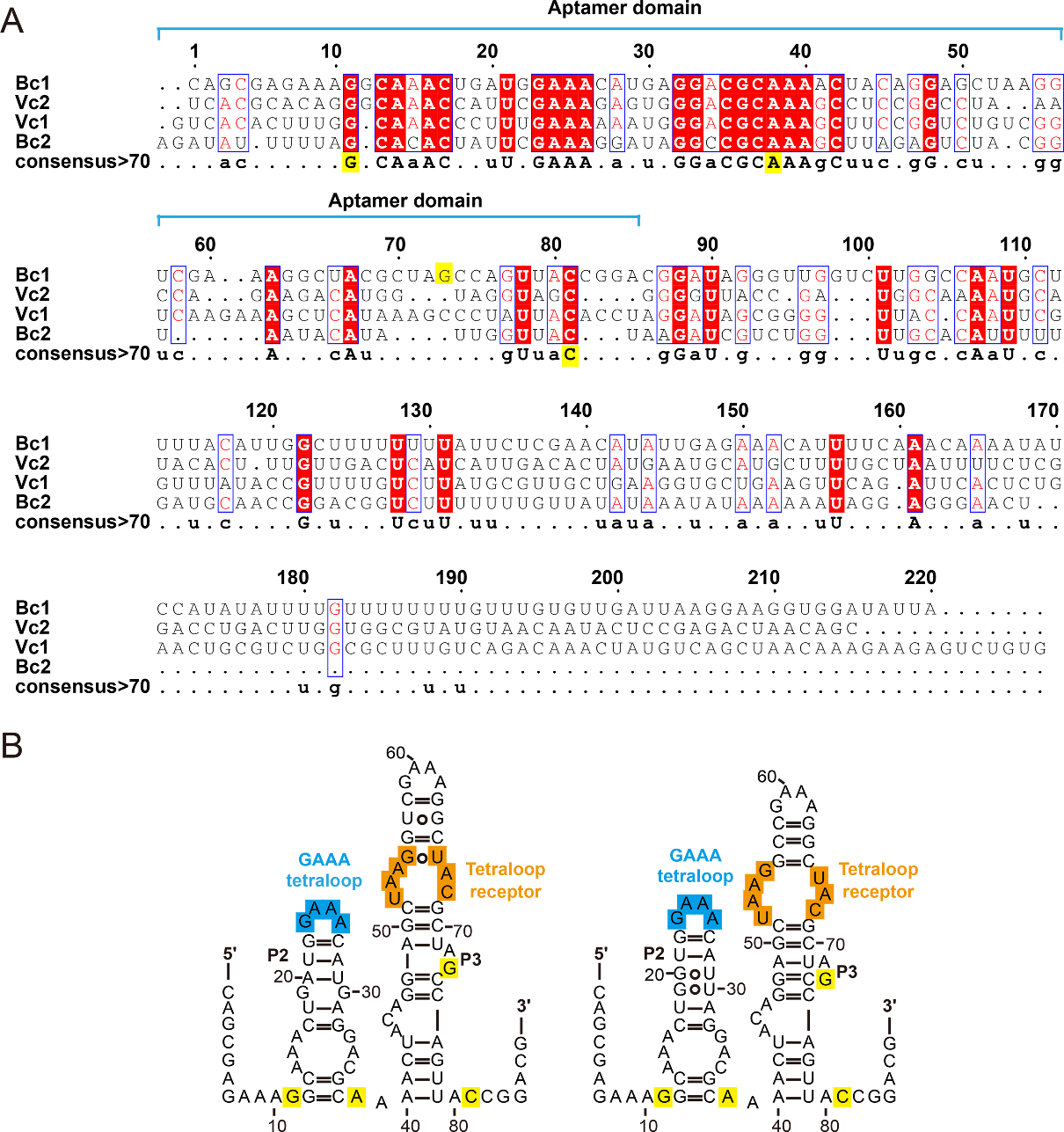


**Supplementary Figure S2. The Sequence and simulated secondary structure of Bc1 are conserved, related to Fig. 1.**

(A) Sequence alignment analysis of Bc1 with other c-di-GMP-I riboswitches Bc2, Vc1, and Vc2. ClustalW 2.1 was used for sequence comparison, and ESPript 3.0 for sequence display. The aptamer domain of Bc1 is indicated by blue lines. Red background indicates completely conserved nucleotides between Bc1 and the other 3 c-di-GMP-I riboswitches, which are emphasized with capital letters, and nucleotides with >70% conservation are indicated with lowercase letters. Letters highlighted in yellow indicate the potentially important nucleotides G11, A38, G73, and C81 of Bc1. (B) Schematic secondary structures of the Bc1 aptamer domain of *B. thuringiensis* BMB171 (left) and *B. cereus* 6A15 (ATCC 10987) (right). Letters highlighted in blue and orange represent the GAAA tetraloop and the corresponding tetraloop receptor of the c-di-GMP-I type I riboswitch, respectively. Letters highlighted in yellow indicate the three conserved nucleotides G11, A38, G73, and C81 that may be essential for Bc1. The Bc1 nucleotide numbers were annotated according to the reported Vc2 riboswitch sequence (7).


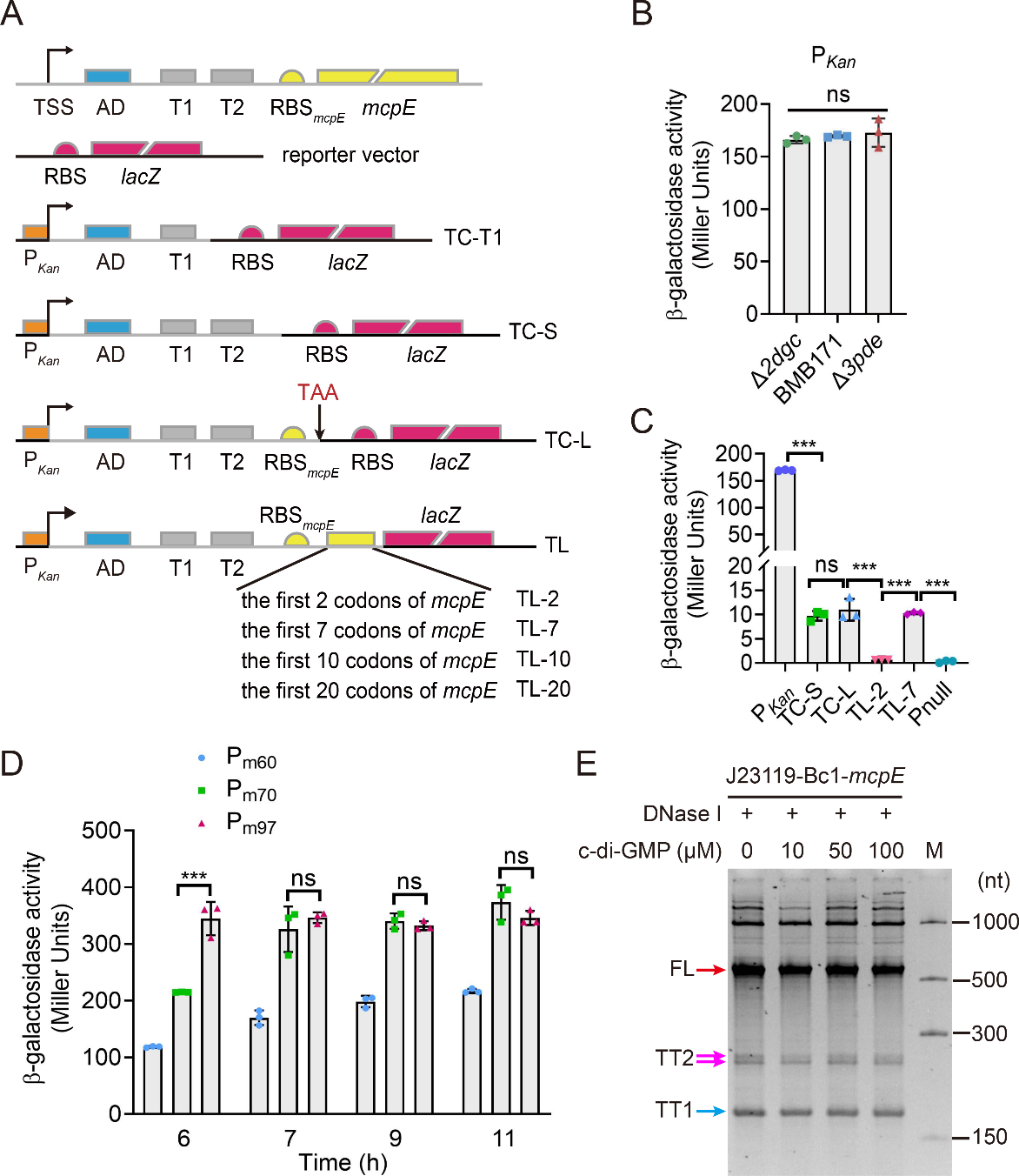


**Supplementary Figure S3. The Bc1 intrinsic dual terminator inhibits downstream gene expression, related to Fig. 2.**

(A) Schematic diagram of the *mcpE* gene structure and various transcriptional and translational fusion constructs. TC: transcriptional fusion, TL: translational fusion. BMB171 genome sequence is shown in grey. Bc1 AD, the two terminators and the *mcpE* gene including RBS is indicated in blue, grey and yellow, respectively. The exogenous promoter P*_Kan_* from *B*. *subtilis* Bs168 is shown in orange. The sequence of the reporter plasmid is shown in black, and its reporter *lacZ* gene and RBS are shown in magenta. TC-T1, TC-S, TC-L denote the reporter constructs that *lacZ* was transcriptionally fused with P*_Kan_* and different lengths of 5' UTR coding sequence up to terminator T1, two terminators, or *mcpE* RBS, respectively. In TC-L, an extra "TAA" is inserted between the coding sequence of the *mcpE* RBS from BMB171 and the RBS of the *lacZ* gene, as indicated by the black straight arrow. TL-2, TL-7, TL-10, and TL-20 denote reporter constructs that the *lacZ* is translationally fused with P*_Kan_* and the coding sequence of the entire 5' UTR up to the first 2, 7, 10, or 20 codons of *mcpE*, respectively.

(B) β-Galactosidase activity assay of Δ*2dgc*, BMB171, Δ*3pde* containing the transcriptional reporter construct P*_Kan_* in which *lacZ* is transcriptionally fused to the promoter P*_Kan_* from *B*. *subtilis* Bs168 strain.

(C) β-Galactosidase activity assay of BMB171 containing various transcriptional fusion constructs (such as P*_Kan_*, TC-S, TC-L or P_null_) and translational fusion constructs (such as TL-2, TL-7). P_null_ refers to a reporter construct in which *lacZ* is not transcriptionally fused to any promoter. Values in panels B and C are mean ± standard deviation of three biological replicates, and the significance of the difference was analyzed by one-way ANOVA in GraphPad Prism 8.0.6 with Bonferroni correction. ns means no significance, P>0.05; *P< 0.05; **P<0.01; ***P<0.001.

(D) β-Galactosidase activity assay of BMB171 with various constructs containing transcriptional fusion of the upstream region, such as P_m60_ (-50-+10), P_m70_ (-60-+10), or P_m97_ (-50-+47). Values in panels B, C and D are mean ± standard deviation of three biological replicates, and the significance of the difference was analyzed by one-way ANOVA in GraphPad Prism 8.0.6 with Bonferroni correction. ns means no significance, P>0.05; *P< 0.05; **P<0.01; ***P<0.001.

(E) *In vitro* transcription termination assay initiated by the J23119 promoter. M denotes the ssRNA marker, whose bands are indicated on the left as 1000, 500, 300, and 150 nt. Full-length transcripts (FL) are indicated by red arrows, and truncated transcripts terminated by T1 (TT1) or T2 (TT2) are indicated in blue or carmine arrows, respectively. Working concentrations of c-di-GMP are 0, 10, 50 and 100 μM.


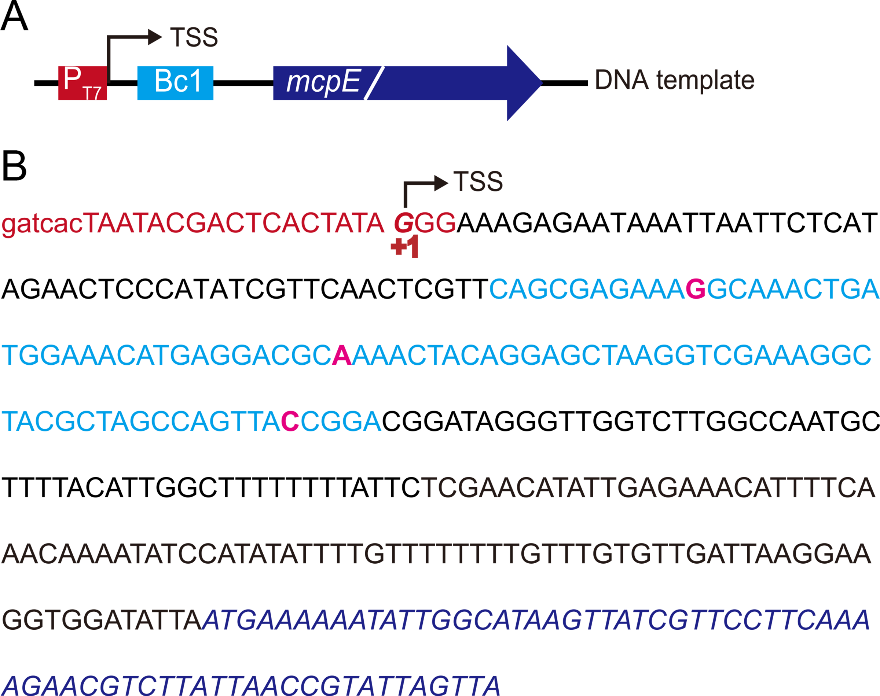


**Supplementary Figure S4. *In vitro* transcription template for the SHAPE-MaP assay, related to Fig. 4.**

(A) Schematic of the *in vitro* transcription template for the SHAPE-MaP assay. The coding sequences of the T7 promoter, Bcl AD, and *mcpE* gene are shown in red, light blue, and dark blue, respectively.

(B) DNA template sequence for *in vitro* transcription in SHAPE-MaP. The color of the coding sequence for each element corresponds to the color in Figure S4A. TSS, +1 is indicated by bold red italic G.


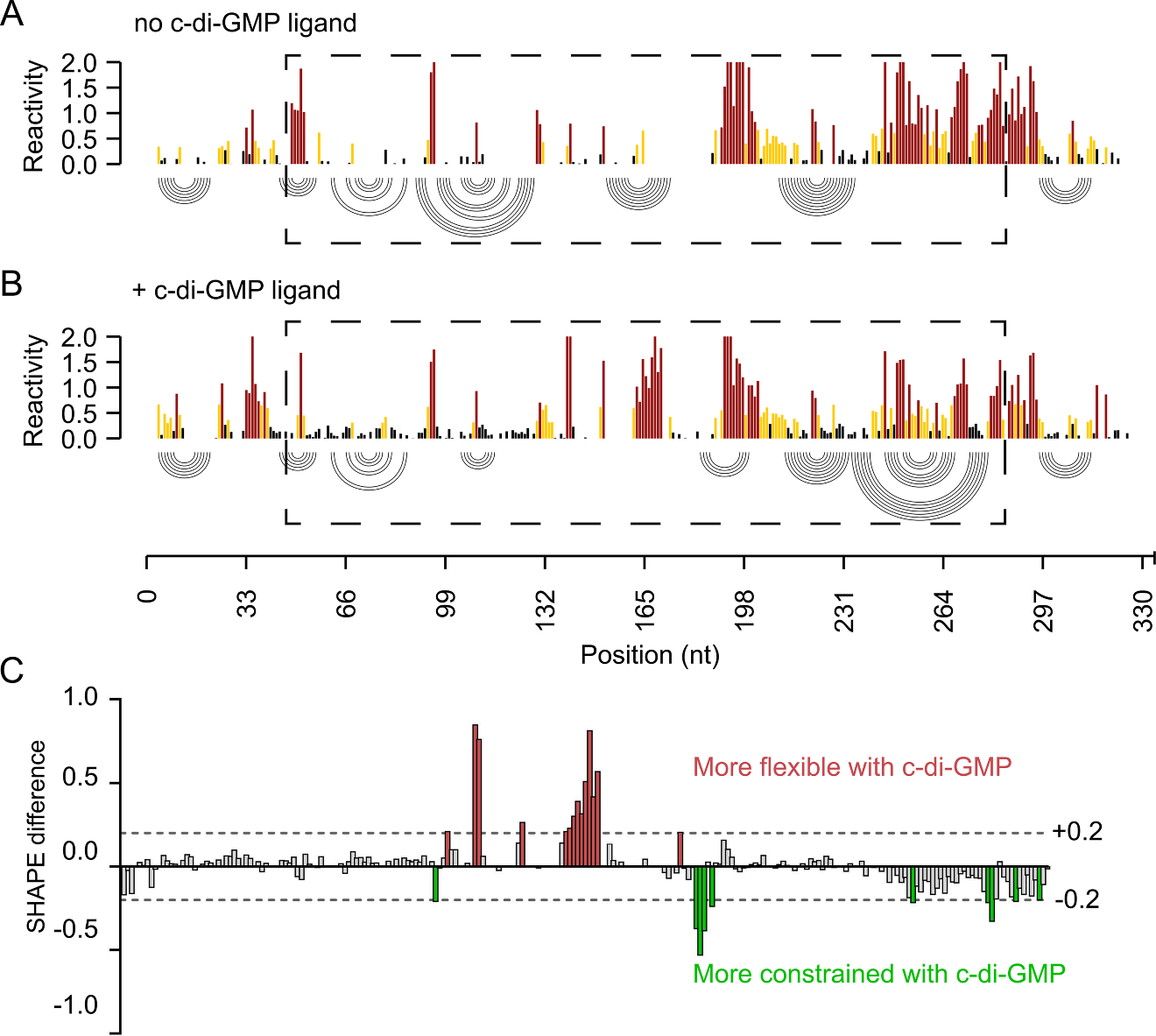


**Supplementary Figure S5.** **Changes in normalized SHAPE-MaP reactivity values for Bc1-containing transcripts upon c-di-GMP binding, related to Fig. 4.**

(A) Normalized SHAPE-MaP reactivity values for Bc1-containing transcripts in absence of c-di-GMP.

(B) Normalized SHAPE-MaP reactivity values for Bc1-containing transcripts in presence of c-di-GMP. The black dashed box in A and B indicates the transcribed region 51-285 where the reactivity values change significantly upon c-di-GMP addition.

(C) SHAPE difference refers to the difference in reactivity values after c-di-GMP addition. Taking -0.2 and 0.2 as references, red indicates residues with more constrained interactions with c-di-GMP, while green indicates residues with more flexible interactions with c-di-GMP.


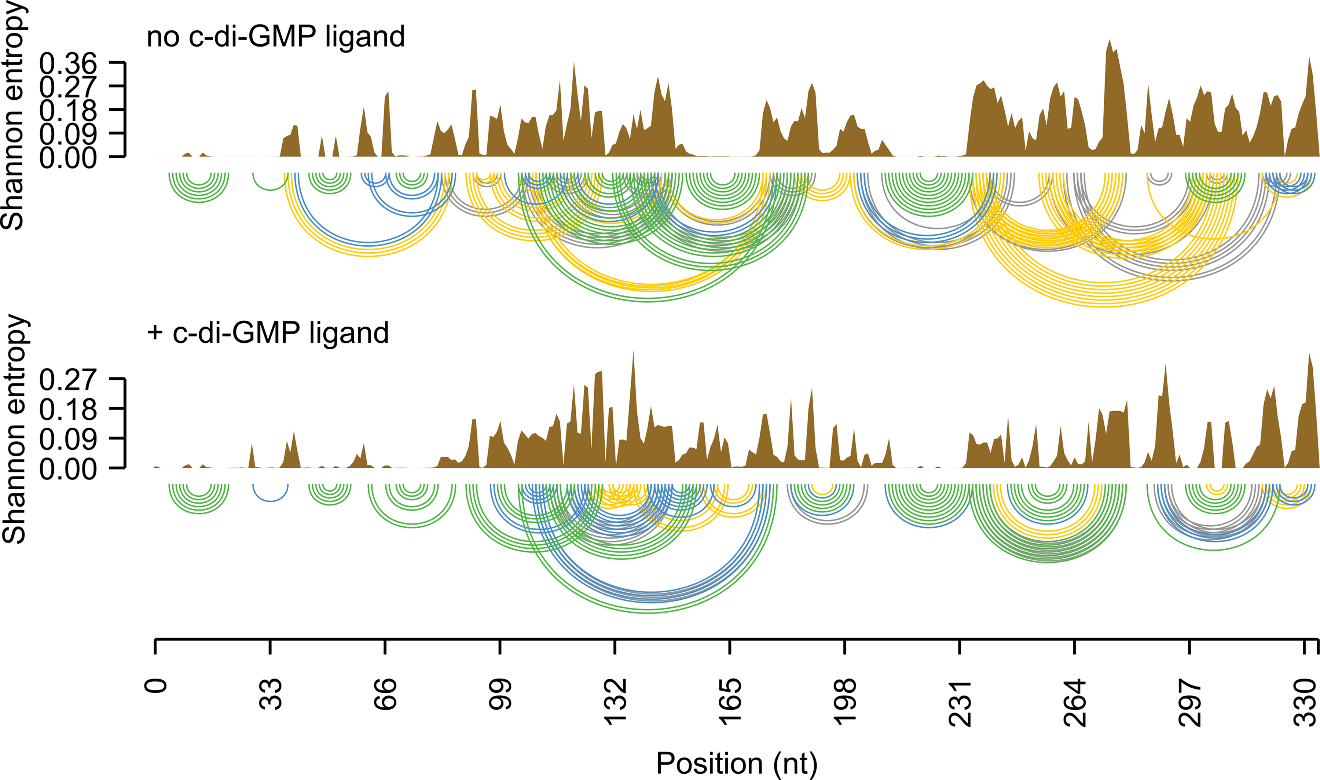


**Supplementary Figure S6. Shannon entropy values for Bc1-containing transcripts in SHAPE-MaP in the absence (*top*) or presence (*bottom*) of c-di-GMP, related to Fig. 4.**

**
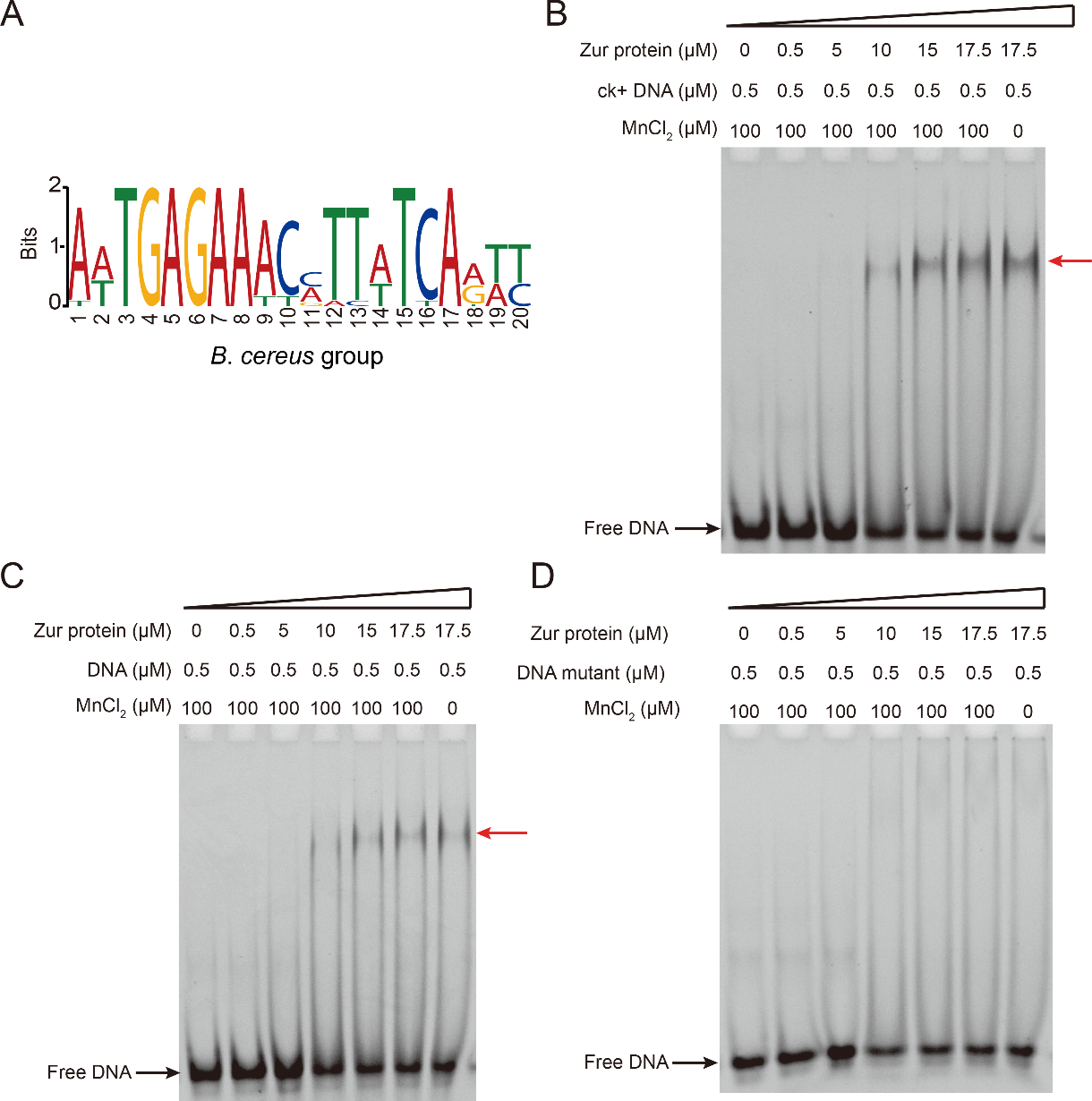
**

**Supplementary Figure S7. Zur transcription factor inhibits Bc1 response to c-di-GMP, related to Fig. 6A.**

(A) Conservation analysis of putative motifs for Fur family transcription factor binding in the Bc1 dual terminator coding sequences of 16 bacteria from the *B*. *cereus* group.

(B) EMSA detects direct interaction of the transcription factor Zur with the positive control sequence (ck+). ck+ DNA is derived from the 50 nt *dhbA* gene (ttatttttataattGATAATGATAATCATTATCaatagattgcgtttttc), which is negatively regulated by Fur_Bs_ (Fur of *B*. *subtilis*) (8).

(C) EMSA detects direct interaction of Zur with the of Bc1 dual terminator coding sequence.

(D) EMSA detects direct interaction of Zur with the mutant Bc1 dual terminator coding sequence. Red and black arrows in B, C and D indicate shifted DNA bands or non-shifted free bands, respectively.

**Supplementary References**

1. Janes BK, Stibitz S. 2006. Routine markerless gene replacement in *Bacillus anthracis*. Infect. Immun 74:1949–1953. <https://doi.org/10.1128/IAI.74.3.1949-1953.2006>.
2. Altenbuchner J. 2016. Editing of the *Bacillus subtilis* genome by the CRISPR-Cas9 system. Appl Environ Microbiol 82:5421-5427. <https://doi.org/10.1128/AEM.01453-16>.
3. Tan TT, Zhang XD, Miao Z, Yu Y, Du SL, Hou XY, Cai J. 2019. A single point mutation in *hmgA* leads to melanin accumulation in *Bacillus thuringiensis* BMB181. Enzyme Microb Technol 120:91-97. <https://doi.org/10.1016/j.enzmictec.2018.10.007>.
4. He J, Shao X, Zheng H, Li M, Wang J, Zhang Q, Li L, Liu Z, Sun M, Wang S, Yu Z. 2010. Complete genome sequence of *Bacillus thuringiensis* mutant strain BMB171. J Bacteriol 192:4074-4075. <https://doi.org/10.1128/JB.00562-10>.
5. Fu Y, Yu Z, Liu S, Chen B, Zhu L, Li Z, Chou SH, He J. 2018. c-di-GMP regulates various phenotypes and insecticidal activity of Gram-positive *Bacillus thuringiensis*. Front Microbiol 9:45. <https://doi.org/10.3389/fmicb.2018.00045>.
6. Tang Q, Yin K, Qian H, Zhao Y, Wang W, Chou SH, Fu Y, He J. 2016. Cyclic di-GMP contributes to adaption and virulence of *Bacillus thuringiensis* through a riboswitch-regulated collagen adhesion protein. Sci Rep 6:28807. <https://doi.org/10.1038/srep28807>.
7. Sudarsan N, Lee ER, Weinberg Z, Moy RH, Kim JN, Link KH, Breaker RR. 2008. Riboswitches in eubacteria sense the second messenger cyclic di-GMP. Science 321:411-413. <https://doi.org/10.1126/science.1159519>.
8. Baichoo N, Wang T, Ye R, Helmann JD. 2002. Global analysis of the *Bacillus subtilis* Fur regulon and the iron starvation stimulon. Mol Microbiol 45:1613-1629. <https://doi.org/10.1046/j.1365-2958.2002.03113.x>.
